# Relationships between farmer well-being and the welfare of their animals: A One Welfare scoping review

**DOI:** 10.1017/awf.2025.10056

**Published:** 2026-01-15

**Authors:** Pierre Levallois, Sebastien Buczinski, Marion Desmarchelier, Sonia Lupien, Marianne Villettaz Robichaud

**Affiliations:** 1 Department of Clinical Sciences, Faculty of veterinary medicine, University of Montreal, 3200 Rue Sicotte, Saint-Hyacinthe, QC, J2S 2M2, Canada; 2 Centre for Studies on Human Stress, Research Center of the Montreal Mental Health University Institute, 7331 Rue Hochelaga, Montréal, QC, H1N 3V2, Canada; 3 Department of Psychiatry and Addiction, Faculty of medicine, University of Montreal, 6128 Succursale Centre-Ville, Montréal, QC, H3C 3J7, Canada

**Keywords:** Animal welfare, assessment, human-animal, mental health, One Health, sustainable agriculture

## Abstract

Although there are public expectations regarding improvements to farm animal welfare, farmers’ well-being remains largely overlooked. This is particularly concerning given the high prevalence of physical and mental health issues among farming populations. As key stakeholders in the implementation of animal welfare practices, farmers play an essential role in welfare outcomes. Improving animal welfare may require addressing farmers’ own well-being. To support this hypothesis, it is necessary to examine the relationship between farmers’ well-being and the welfare of their animals. This scoping review aimed to: (1) map the methods used to describe relationships between farmer well-being and animal welfare in primary research; and (2) compile pieces of evidence of such relationships. Following the PRISMA extension for Scoping Reviews, the same search was carried out on three databases (Web of Science Core Collection, MEDLINE, CABI digital library). Twenty-two articles from the 10,189 retrieved met the inclusion criteria. Results underscored the need to standardise methods to enable cross-study comparisons, as different questionnaires were used to assess the same construct (e.g. four for psychological stress), and none of the animal welfare indicators were fully comparable. Moreover, 94 pieces of evidence regarding the relationships between farmer well-being and the welfare of their animals were compiled. Ninety-three pieces described positive associations where improved farmer well-being was associated with improved welfare of their animals, and *vice versa.* This result suggests that welfare improvement strategies on farms should address not only animal welfare, but also farmer well-being. The results therefore support a One Welfare approach on commercial farms.

## Introduction

Improving farm animal welfare is currently an expectation from a section of the public (Clark *et al.* 2016; Alonso *et al.* 2020). Animal welfare can be scientifically defined as “*the positive mental and physical state linked to the satisfaction of its physiological and behavioural needs, as well as its expectations. This state varies according to the animal’s perception of the situation*” (Anses 2018; p 16). Although farm animal welfare awareness varies with socio-demographic characteristics, such as age, gender, education and location (urban or rural), the notions of naturalness and humane treatment have been reported as central constituents to public acceptability (Clark *et al.* 2016). These notions could explain why specific management practices or topics are concerning for a part of the public, as ensuring outdoor access for animals or managing ‘surplus’ dairy calves (Hötzel *et al.* 2017; Wilson *et al.* 2020). Meeting public expectations such as these contributes to address the social sustainability of farm animal industries (Bolton *et al.* 2024). Indeed, when farming practices align with public values, consumers are more likely to continue purchasing animal-based products (Clark *et al.* 2017). Nonetheless, willingness-to-pay for higher standards of animal welfare vary across people, and have been reported to decrease with social characteristics like age and incomes (Lagerkvist & Hess 2011; Clark *et al.* 2017). This attitude-behaviour gap could pose a challenge when it comes to financing improvements in animal welfare. Indeed, addressing public expectations by improving farm animal welfare requires time and money investments from farmers (Grethe 2017). Farmers need to learn to carry out new practices, invest in new equipment, face new challenges, or build new housing to comply with welfare recommendations and regulations (Sumner *et al.* 2010; NFACC 2023). As a result, farmers play a key role in improving animal welfare and addressing public expectations.

Yet, farmer well-being is rarely considered within public expectations (although no consensual definition seems to exist, an individual’s well-being can be defined as “*the state of being healthy, happy, or prosperous; physical, psychological, or moral welfare*” [Oxford English Dictionary 2023]). Furthermore, practices favouring farmer well-being have been reported to be valued less by the public than those favouring animal welfare. By way of example, “*All cattle must have access to outdoor exercise areas for at least 4 hours per day, weather permitting*” was considered to be more important than “*All workers are provided paid 15-minute breaks for every 4 hours worked, and a 30-minute* [meal] *break between each 4-hour shift*” (Kaminski *et al.* 2024; Tables 1 and 3, respectively, pp 26 and 31). The existence of a lower consideration towards farmer well-being is concerning, given that physical and mental health disorders are over-represented in the farming population. Regarding physical health, over-representations of respiratory attack related to work, and long-standing musculoskeletal pain have been reported (respectively compared to skilled manual collar, and skilled white collar; Steen *et al.* 2023). Regarding mental health, over-representations of symptoms of psychological stress, anxiety, depression, burn-out, suicidal ideation, and suicide have been described in farming populations (Kallioniemi *et al.* 2016; Jones-Bitton *et al.* 2020; Steck *et al.* 2020; O’Shaughnessy *et al.* 2022; Montgomery *et al.* 2024).

Improving animal welfare may require addressing farmer well-being. Farmers themselves considered taking care of their own well-being as the most important and influential way to improve animal welfare (Kauppinen *et al.* 2013). On one hand, farmers experiencing poor well-being may struggle to improve the welfare of their animals, as they may not be physically, mentally or emotionally available to do so. In such circumstances, asking farmers to address public expectations for improved animal welfare could be particularly challenging. On the other hand, improved farmer well-being may foster more positive attitudes toward animals. Positive attitudes are associated with improved management practices and animal welfare outcomes, such as less fearful cows, lower prevalences of carpus skin lesions and lameness in ewes at mid-pregnancy (Kielland *et al.* 2010; des Roches *et al.* 2016; Munoz *et al.* 2019). Besides, improving animal welfare can be mentally rewarding for farmers (Kauppinen *et al.* 2010). A positive association between farmer well-being and the welfare of their animals may therefore exist. To support this hypothesis, it is necessary to examine and better understand the relationships between farmer well-being and the welfare of their animals. If evidence supports this hypothesis, it could not only highlight the importance of addressing farmer well-being but also help to identify leverage points to improve animal welfare.

The One Welfare approach provides a framework to examine the relationships between farmer well-being and the welfare of their animals. One Welfare “*serves as a call to recognise the many interconnections between human* [well-being]*, animal welfare and the integrity of the environment*” (Pinillos *et al.* 2016). Although conceptually close to the One Health approach, it completes it by explicitly integrating welfare considerations (WHO 2023). The One Welfare approach is relatively recent, having been first introduced in 2014 (OneWelfare 2023).

As the One Welfare approach is recent, methods for examining the relationships between farmer well-being and the welfare of their animals are potentially still being explored. In particular, a range of methods can be used to assess both farmer well-being and animal welfare. For instance, different audits with distinct items and scoring methods can be used to assess dairy cattle welfare alone (Welfare Quality® 2009; Dairy Farmers of Canada 2023). Such differences in methods limit direct cross-study comparisons. However, comparable results are essential to determine whether findings align. Mapping the methods used to describe the relationships between farmer well-being and the welfare of their animals could support future study designs and contribute to enhance cross-study comparability. Indeed, such a mapping would provide an inventory of methods used to date (including questionnaires, audits or tools used, indicators calculated, and data analyses conducted) and allow recommendations to be suggested, addressing methodological gaps and limitations.

A scoping review is “*a type of evidence synthesis that aims to systematically identify and map the breadth of evidence available on a particular topic*” (Munn *et al*. 2022). Unlike a systematic review followed by meta-analysis, a scoping review does not aim to determine the effect of an intervention, nor to critically appraise individual evidence sources. Instead, it allows a qualitative examination of “*how research is conducted on a certain topic or field*”, and to “*identify the types of available evidence in a given field*” (Munn *et al.* 2022). A scoping review seems thus well-suited to map the methods used so far to examine the relationships between farmer well-being and the welfare of their animals, as well as to compile potential pieces of evidence of such relationships. These pieces could inform discussion regarding whether a positive relationship exists between farmer well-being and the welfare of their animals. To date, scoping reviews were carried out to investigate the effect of farmers’ personality and attitudes on dairy cattle welfare (Adler *et al.* 2019), and the role of human-animal relationships in dairy cattle welfare (Adamczyk 2018). To the best of our knowledge, no scoping reviews have been carried out to explore the relationships between farmer well-being and the welfare of their animals on commercial farms.

The primary aim of this scoping review was to identify the methods used to describe the relationships between farmer well-being and the welfare of their animals in primary research articles. Specifically, the review focused upon the questionnaires or tools used, indicators measured, and data analyses conducted. The secondary aim was to compile results of relationship descriptions between the farmer well-being and the welfare of their animals from the articles included in the scoping review.

## Materials and methods

### Protocol

The protocol for this scoping review was developed in accordance with the PRISMA extension for Scoping Reviews (PRISMA-ScR; Tricco *et al*. 2018), and guided by the recommendations of Peters *et al*. (2022). The protocol was developed before starting the study and was pre-registered at the following repository: https://doi.org/10.5281/zenodo.13284408 (Levallois *et al.* 2024).

### Eligibility criteria

Primary research articles of any study design were eligible for inclusion, provided they included the relationship description between at least one farmer well-being indicator, and at least one indicator of their farm animals’ welfare.

#### Farmer well-being

To be eligible, a farmer well-being indicator had to meet two criteria. First, it had to measure a state related to one of the five well-being dimensions mentioned below. These dimensions were conceptualised in this review to allow for a precise mapping of the indicators used to assess farmer well-being. According to the Oxford English Dictionary (2023), individual well-being is defined as “*the state of being healthy, happy, or prosperous; physical, psychological, or moral welfare*”. That is why it was considered that a farmer well-being indicator had to measure a state, related to farmers’ (1) mental health, (2) physical health, (3) satisfaction towards a life aspect (so that more aspects than only prosperity could be considered), or (4) emotion (to include more emotions than just happiness). A fifth category, feelings, was added to distinguish emotions from feelings. This distinction was based upon the idea that an emotion has a ‘rapid onset, short duration’, whereas a feeling is the cognitive integration of an emotion having a longer duration (Ekman 1992; Damasio 1995). Including this distinction enabled more nuanced mapping of the well-being indicators. The term ‘farmer well-being dimensions’, as used throughout this review, refers to these five categories as distinct aspects of well-being. Second, a farmer well-being indicator had to be measured at a farmer-level, as the review focuses on the relationships between individual farmer well-being and the welfare of their animals. Measures of farmer well-being indicators conducted at broader scales, like a population scale, were excluded because they do not specifically inform about one farmer. Of note, a distinction was made between assessing a farmer well-being state and assessing the perceived farmer well-being importance. The latter was not eligible as a farmer well-being indicator in this review, as it does not reflect an actual well-being state.

### Animal welfare

Only livestock and poultry were considered (animals from aquaculture were excluded; for more details regarding the animals considered, see Table 1). To include a potential diversity of livestock and poultry species, no geographical restrictions were applied to the search strategy.

**Table 1.** Search strings used to retrieve primary research articles where the relationships between farmer well-being, and the welfare of their animals was assessed

String	Keywords
**1**	farmer* OR producer* OR rancher*
**2**	animal OR cow* OR calf OR calves OR veal OR cattle OR bovine OR beef OR steer* OR pig* OR sow OR boar* OR swine OR chicken OR turkey OR duck* OR goose OR geese OR poultry OR horse OR donkey OR equine OR sheep OR lamb* OR goat OR rabbit OR deer OR camel* OR buffalo* OR llama OR alpaca OR yak* OR mink* OR ruminant OR monogastric OR livestock OR herd* OR flock
**3**	wellbeing OR well-being OR welfare
**4**	associat* OR relat* OR correl* OR link*
**Final search**	1 AND 2 AND 3 AND 4

To be eligible, an animal welfare indicator had to meet two criteria. First, it had to measure a state, or an aspect related to one of the six welfare dimensions mentioned below. As with farmer well-being, these dimensions were conceptualised to enable a precise mapping of the indicators used to assess animal welfare. According to Anses (2018; p 16), animal welfare “*is the positive mental and physical state linked to the satisfaction of its physiological and behavioural needs, as well as its expectations. This state varies according to the animal’s perception of the situation*”. Based on this definition, an animal welfare indicator had to measure a state, with an animal-based measure related to physiology, behaviour or physical health. In addition, although they do not inform about a welfare state, measures related to housing environment and management practices were also deemed eligible. They indeed provide insights into the animals’ living conditions. Six categories, based on the five domains described by Mellor and Beausoleil (2015) with the addition of farm management, were considered to classify the animal-based, environmental-based, and management-based measures: (1) nutrition, (2) environment, (3) health, (4) behaviour, (5) mental state, and (6) management (practices unrelated to nutrition, environment and health, e.g. handling to move animals). As with farmer well-being dimensions, the term ‘animal welfare dimensions’ used hereunder refers to these six categories. Second, an animal welfare indicator had to be measured at an animal-, herd-, or farm-level. Of note, production indicators, like average daily gain or milk production per year, were not considered as welfare indicators, as they do not directly inform about a welfare state, nor the life conditions of the animal. Likewise, assessments of the perceived importance of animal welfare were excluded, as they do not inform about the actual welfare state or animals’ living conditions.

Besides these criteria, there were no date nor language restrictions. An article written in a language other than English was translated using the file translation feature in DeepL (DeepL SE, Cologne, Germany; https://www.deepl.com/en/translator).

### Information sources

Three databases were used for the literature search: Web of Science Core Collection (https://www.webofscience.com), MEDLINE (https://pubmed.ncbi.nlm.nih.gov), and CABI digital library (https://www.cabidigitallibrary.org). The search was conducted using the full range of publication dates available in each database.

### Literature search

The final search was completed on September 3^rd^, 2024, by two independent reviewers (PL and MVR). The final search is detailed in Table 1 and was adapted to the format requirements of each database. The final search was conducted on the Web of Science Core Collection considering all fields, MEDLINE considering only the “title/abstract” field, and CABI digital library considering only the “abstract” field for content to which we had access. References retrieved from the three databases were exported and merged into a single Excel® file (Microsoft®, Redmond, WA, US). Deduplication was then carried out on the Excel® file in three steps before title and abstract screening: (1) references which had a DOI were deduplicated based on DOI information; (2) all references were then deduplicated based on reference titles; (3) remaining duplicates were manually removed during the initial screening based on titles and abstracts.

Of note, the use of the asterisk “*” in strings 1 and 2 was pre-tested on the Web of Science Core Collection (Table 1). For each keyword (from ‘farmer’ in string 1, to ‘flock’ in string 2; excluding keywords without suffixes such as ‘calves’, ‘veal’, or ‘cattle’), the number of search results was observed with and without the asterisk. If adding the asterisk did not affect the number of the results, it was removed from the final search string.

### Selection of sources of evidence

Sources of evidence were selected in two steps: (1) title and abstract screening, followed by (2) full-text screening. Both steps were conducted independently by two reviewers (PL and MVR). Discussions between both reviewers regarding the selection of sources of evidence occurred only twice: at the end of each step.

The screening questions are detailed in the pre-registered protocol (Levallois *et al.* 2024). Briefly, these questions were designed to assess the following eligibility criteria: (1) the article was a primary research study; (2) it examined the relationship between at least; (3) one farmer well-being indicator; and at least (4) one welfare indicator of their farm animals; (5) in livestock or poultry species. Of note, precisions were added after protocol registration to two questions regarding the full-text screening. It specified that the farmer well-being and animal welfare indicators had to relate to their respective dimensions as previously defined.

At the end of each step, any disagreement regarding the inclusion of a study in the next phase of the review was resolved by consensus, following consultation with a third author (SB). Reasons for exclusions at the second step were recorded.

### Data charting process

The selection and inclusion process was recorded according to the Preferred Reporting Items for Systematic Review and Meta-analysis (PRISMA) flow diagram proposed by Moher *et al*. (2009). An Excel® spreadsheet was created to compile the retrieved items from the included articles.

### Data extraction

Data extracted from the articles included in the scoping review concerned nine main categories, as outlined in the pre-registered protocol (Levallois *et al.* 2024). Additional data not specified in the pre-registered protocol were also extracted. Details regarding the rationale for including these additional data, as well as how they were recorded, are provided below the itemised list. Briefly, the (1) study characteristics, (2) authors affiliation, (3) objective and hypotheses of the study, (4) study design, (5) animal genus and breed studied, and (6) total number of participants to the study (plus the justification of respective sample sizes, when available) were extracted. Moreover, the method(s) used to assess (7) farmer well-being and (8) animal welfare (including for both: questionnaire or tool used, dimension concerned, aspect of the dimension assessed, indicators measured or calculated from the data collection), as well (9) to describe the relationships between the farmer well-being indicator(s), and animal welfare indicator(s) (data transformation of measured or calculated indicators, statistical analysis in quantitative studies, derivation of themes in qualitative studies) were extracted. Of note, the ENTREQ guidelines was considered to extract methodological information from qualitative studies (Tong *et al.* 2012; notably regarding the methodological rationale, i.e. framework, model, philosophy, and derivation of themes). Finally, (10) the results of the relationship descriptions between the farmer well-being indicator(s) and animal welfare indicator(s) were compiled. Each positive relationship that was statistically significant in quantitative studies, or reported in qualitative studies, was considered as a piece of evidence. The nature of extracted results differed between quantitative and qualitative studies:

- Quantitative study: statistical indicator used to examine a relationship between the farmer well-being and animal welfare indicators (based on the data analysis and result sections of the publications); value of the statistical indicator describing a such relationship; *P*-value.
- Qualitative study: quotes where the presence (or absence) of a relationship between indicators of the farmer well-being and the welfare of their animals was explicitly mentioned (i.e. when wording directly referred to well-being or welfare dimensions or aspects thereof). If the same relationship between two indicators was mentioned multiple times in the same article: (1) only one illustrative quote was extracted, and (2) the relationship was counted as a single piece of evidence.

The authors’ affiliations were examined in more detail to determine whether each study involved collaboration between researchers from human and animal science departments in describing potential relationships between farmer well-being and the welfare of their animals. Such interdisciplinary collaboration may be required for adequately examining these relationships. Authors’ affiliation was recorded as stated in the articles. An author was classified as affiliated with a human science department when the affiliation included: “public health” or “medicine” (excluding veterinary), “psychology”, “social” or “sociology”. An author was classified as affiliated with an animal science department when the affiliation included: “animal”, “beef”, “dairy”, “life sciences”, “veterinary” or “wildlife”. An author was classified as affiliated with a department other than human or animal science when the affiliation included: “economics”, “environment” or “statistics”. Sometimes, the affiliation wording was not specific, including: “agricultural research”, “farm advisory service”, “university of [town or country]”, “scientific research”. When it occurred, these affiliations were coded as “unknown” to avoid mis-recording. To ensure accuracy, all affiliations classified as human or animal science department (or unknown) were double-checked by consulting the websites of the related research departments.

Assignment to a farmer well-being dimension was justified by precising which aspect of the dimension was assessed, i.e. for (1) mental health: stress, anxiety, depression, burn-out, somatisation, worries, trauma, resilience; (2) physical health: pain, injury, sickness; (3) satisfaction: life in general, work, standard of living, future, social relationships, environment; (4) emotion: anger, disgust, fear, happiness, sadness (Ekman 1999); (5) feeling: bewilderment, frustration, hopelessness, loss of confidence, miserability, optimism, relaxation, self-doubt, shame, usefulness.

Assignment to an animal welfare dimension was similarly justified by precising which aspect of the dimension was assessed, i.e. for (1) nutrition: feed and water intakes; (2) environment: housing comfort (thermal, air, space for movements); (3) health: body condition, injury, mortality, specific disease or disorder related to digestion, locomotion, metabolism, udder health, reproduction, respiration, as well as the prevention and treatment of diseases; (4) behaviour: social behaviours; (5) mental state: emotion, feeling; (6) management: practices implemented not included in the environment, nutrition or health (e.g. animal handling) (Mellor & Beausoleil 2015).

Note that some farmer well-being and animal welfare indicators targeted the dimension in general, without reference to a specific aspect; in such cases, the aspect was labelled as ‘overall’.

Data extraction regarding the nine categories was carried out independently by one reviewer (PL). To ensure consistency, data extraction was pre-tested by both reviewers from five references (PL and MVR). Any disagreements during the extraction process were resolved by consensus, following consultation with a third author (SB).

### Synthesis of the results

Extracted data were synthesised into three summary tables, each focusing on the methods used to (1) assess farmer well-being, (2) assess animal welfare, (3) describe the relationships between both. Quantitative variables, like the number of participants to the study, were described with basic statistics (distribution, mean) using R software version 4.4.0 (R Core Team 2024). For cases in which the number of farms, farmers, or animals was not reported, these studies were excluded from the corresponding calculations, and statistics were computed only from the available data.

Six heat maps were created based on all reported relationships between farmer well-being indicators and animal welfare indicators. Every indicator was related to a dimension and one of its aspects, as mentioned above. The heat maps provided an overview of which (1) dimensions, and (2) aspects thereof of farmer well-being and animal welfare, were the most frequently paired in relationship testing, and which combinations remained unexplored. Six separate heat maps were created to distinguish relationships tested in quantitative (four heat maps in total) and qualitative (two heat maps) studies. Heat maps were generated in Prism version 9.5.0 (Prism, GraphPad Software, Boston, MA, US; graph family = ‘grouped’, graph type = ‘heat map’).

## Results

### Search results

**Figure 1.**
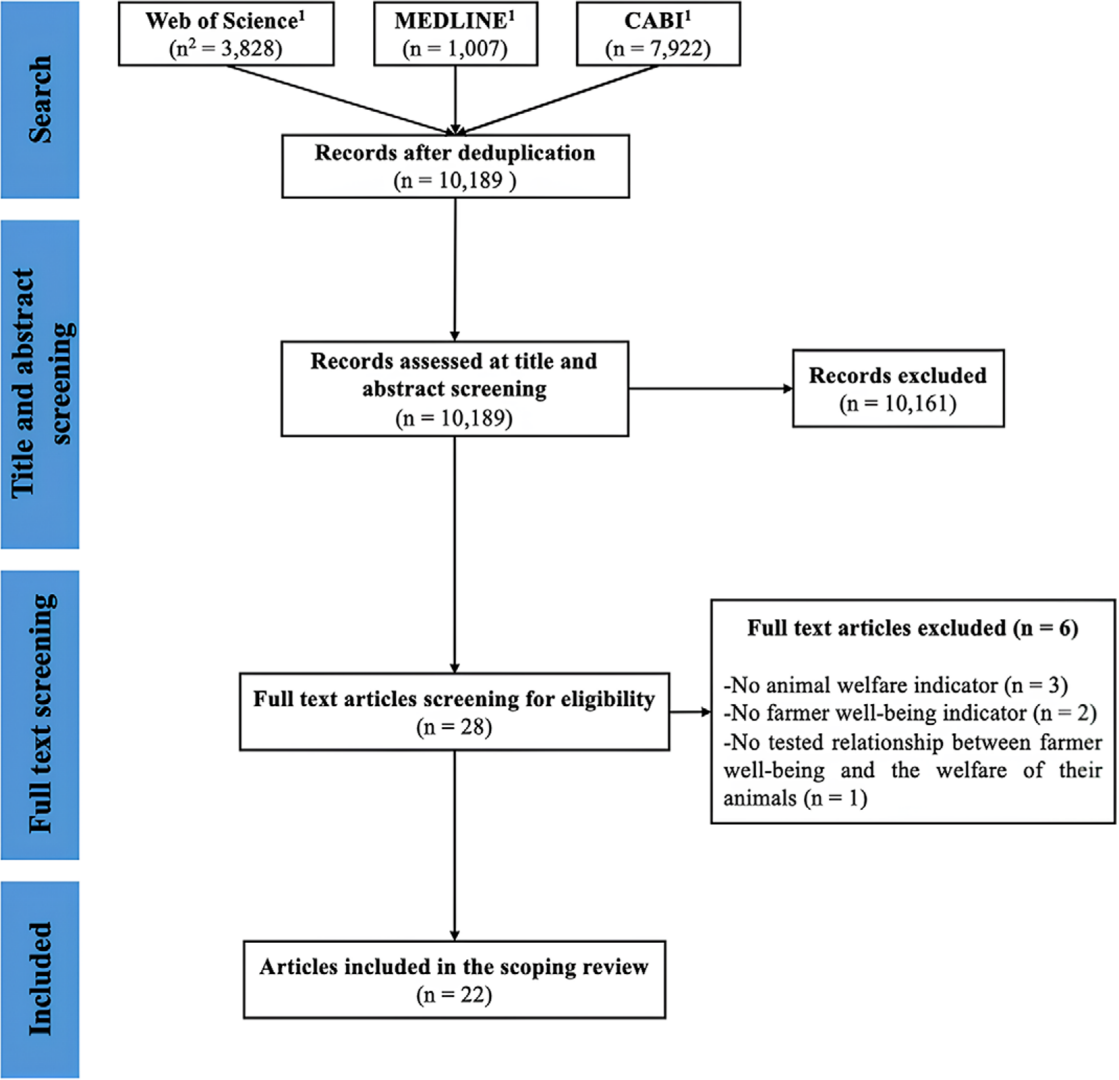
Flow diagram synthesising the results of the search process to retrieve primary research articles regarding the relationships between farmer well-being and the welfare of their animals. N.B. (1) Searches were carried out on Web of Science Core Collection considering all fields, MEDLINE considering only the ‘title/abstract’ field, and CABI digital library considering only the ‘abstract’ field for content we had access; (2) n = number of studies.

The flow diagram presenting the search results is displayed in Figure 1. In total, 10,189 articles were obtained after deduplication for the title and abstract screening, 28 selected for the full text screening and 22 articles were finally included in the review. Of note, there were disagreements for six articles at the end of the first step, and a consensus was obtained consulting the eligibility criteria. There was no disagreement at the end of the second step. There was only one study per article, except for two articles which included results from a mixed-method approach, each including one quantitative and one qualitative study (Crimes & Enticott 2019; Nuvey *et al.* 2020; of note, the qualitative study in both articles did not meet the eligibility criteria since they, respectively, did not include [1] an animal welfare indicator and [2] a farmer well-being indicator). Only the quantitative approach of both articles was eligible for this review. Therefore, only one study was considered in all the included articles. One article was translated from Spanish to English (Medrano-Galarza *et al.* 2023).

### Study-level characteristics

Characteristics of the 22 included articles are described in Table 2. Most of them were published since 2019 (n = 17 studies; 77%). Studies were mainly carried out in Europe (n = 12), then Africa (n = 3), Oceania (n = 3), North America (n = 2), and South America (n = 2). Most of the 22 studies were cross-sectional (n = 18; 82%) and had a quantitative approach (n = 16; 73%). The time of year in which studies were carried out was not mentioned in six articles (27%).

**Table 2.** Description of the study, author, and studied population characteristics regarding the 22 articles included in a scoping review aiming to map the methods used to describe — and compile pieces of evidence of — relationships between farmer well-being and animal welfare

Articles	Study characteristics			Authors affiliation^1^				Population characteristics			
	Country	Study design	Approach	Human	Animal	Other	NA^2^	Genus (*animal*)	N_Farm_^3^	N_Farmer_^3^	N_Animal_^3^
Andreasen *et al.* (2020)	Denmark	Cross-sectional	Quantitative	0	4	0	0	Bovine (*dairy cow*)	35	35	2 415
Calderón-Amor *et al.* (2020)	Chile	Cross-sectional	Quantitative	0	4	0	0	Bovine (*dairy calf*)	28	28	698
Crimes and Enticott (2019)	Wales	Case-control	Quantitative	2	0	0	0	Bovine (*beef, dairy cow*)	NM^4^	582	96,612
de Krom (2015)	Belgium	Cross-sectional	Qualitative	1	0	0	0	Porcine (*sow*)	19	19	4 680
Devitt *et al.* (2015)	Ireland	Cross-sectional	Qualitative	5	0	0	0	Several (bovine: *beef, dairy cow*; ovine: *sheep*)	13	13	NM
Fasina *et al.* (2010)	Nigeria	Case-control	Quantitative	3	3	0	0	Gallus (*poultry*)	104	104	NM
Hansen and Østerås (2019)	Norway	Cross-sectional	Quantitative	0	0	0	2	Bovine (*dairy cow*)	914	914	NM
King *et al.* (2021)	Canada	Cross-sectional	Quantitative	0	3	0	0	Bovine (*dairy cow*)	28	34	2,218
Lee *et al.* (2020)	USA	Cross-sectional	Quantitative	0	9	0	0	Bovine (*dairy cow*)	542	542	110,568
Medrano-Galarza *et al.* (2023)	Bolivia	Cross-sectional	Quantitative	0	0	0	11	Ovine (*ewe, sheep*)	22	22	427
Noller *et al.* (2022)	New-Zealand	Cross-sectional	Qualitative	3	1	0	0	Bovine (*beef, dairy cow*)	18	NM	NM
Nuvey *et al.* (2023)	Ghana	Cross-sectional	Quantitative	5	2	0	1	Several (bovine: *beef, dairy cow*; caprine: *goat*; ovine: *sheep*)	350	350	875
Nuvey *et al.* (2020)	Ghana	Cross-sectional	Quantitative	5	0	0	8	Bovine (*beef, dairy cow*)	287	287	NM
O’Kane *et al.* (2017)	England	Cross-sectional	Quantitative	1	3	0	0	Ovine (*ewe, lamb, sheep*)	1,294	1,294	NM
Perrin *et al.* (2020)	France	Retrospective longitudinal	Quantitative	0	0	4	0	Bovine (*dairy cow*)	81	81	NM
Phythian and Glover (2019)	England	Cross-sectional	Qualitative	0	3	0	0	Ovine (*ewe, lamb*)	6	6	NM
Pol *et al.* (2021)	France	Cross-sectional	Quantitative	0	6	0	0	Porcine (*gilt, sow*)	52	52	NM
Rilanto *et al.* (2022)	Estonia	Cross-sectional	Quantitative	1	7	0	0	Bovine (*dairy cow*)	116	116	59,670
Spigarelli *et al.* (2021)	Italy/Austria	Cross-sectional	Quantitative	0	8	2	0	Bovine (*dairy cow*)	69	69	1,584
Vallance *et al.* (2023)	New-Zealand	Cross-sectional	Qualitative	0	2	1	2	Several (bovine: *beef, dairy cow*; ovine: *sheep*)	10	10	NM
van Calker *et al.* (2007)	Netherlands	Prospective longitudinal	Qualitative^5^	0	1	4	0	Bovine (*dairy cow*)	2	NM	228
Vicic *et al.* (2022)	Australia	Cross-sectional	Quantitative	1	3	1	0	Bovine (*dairy calf*)	127	127	NM

**1:** Authors affiliation = Affiliation to a department of animal sciences, human sciences, other sciences (such as economics [n = 2 researchers], environment [n = 4], or statistics [n = 1]), based on the wording of authors’ affiliations and a double check on the websites related to affiliations. **2:** NA = No Affiliation was recorded since the affiliation wording could be not specific. **3:** N_X_ = Total number of farms, farmers, or animals recruited in the study. **4:** NM = Not Mentioned. The total number of farmers or animals was not explicitly mentioned in five studies (only the median number or quartile distribution was provided) and was not mentioned at all in seven studies. **5:** Qualitative = This study is based on a prospective longitudinal modelling with quantitative data. However, results are only descriptive and no statistical relationships were described. Therefore, the descriptive results were used as if the study had a qualitative approach.

A minority of articles were written by authors affiliated to both animal and human sciences departments (n = 5 studies/18, excluding the four studies with an ‘unknown’ affiliation for this ratio; 28%).

### Study population characteristics

Overall, studies included 4,117 farms (the number of farms was not mentioned in one article), 4,685 farmers (the number of farmers was not mentioned in one article), and 279,945 animals every genus considered (the total number of animals was not explicitly mentioned or mentioned at all in eleven articles). The median numbers of samples per study were 52 farms (interquartile range [IQR] = [19; 127]), 75 farmers (IQR = [27; 303]), and 2,218 animals every genus considered (IQR = [787; 31,389]) (based on the available numbers of farms, farmers, and animals in articles). When mentioned in the 16 quantitative studies, sample sizes were justified with references and statistical calculations only in two studies for farm sample, three studies for farmer sample, and three studies for animal sample. In qualitative studies, sample sizes were convenient (when mentioned). However, the rationale behind the convenience samples was systematically explained in all the six studies and justified with a reference in one study.

**Figure 2.**
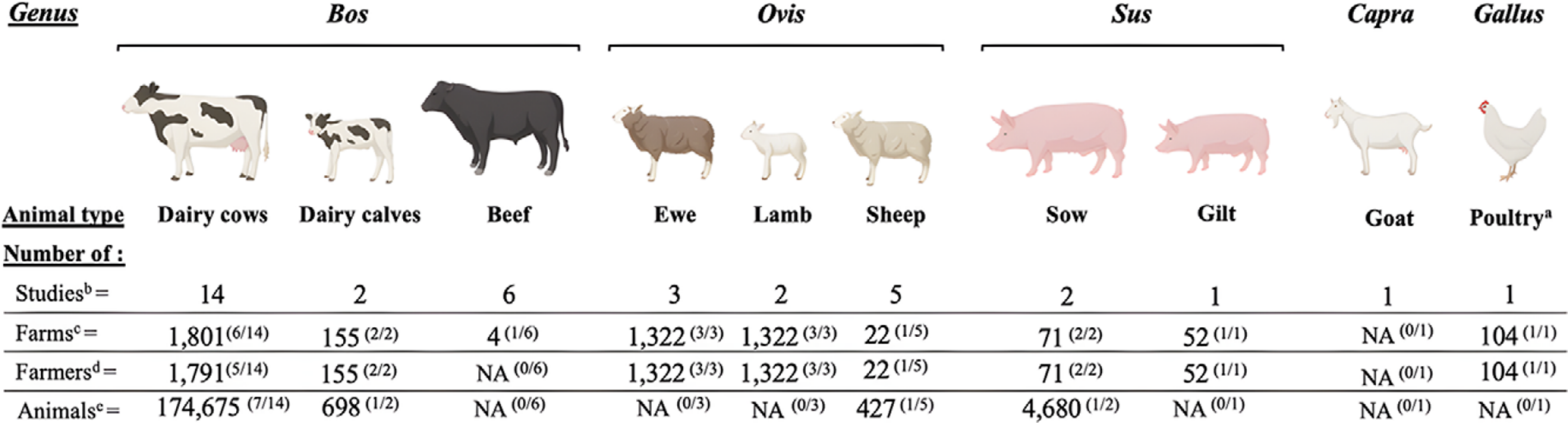
Description of the samples recruited in the 22 reviewed studies according to animal types. N.B. (a) the only study focusing on poultry included both chicken and egg-laying hen farms, (b) total number of studies in which the type of animals was included (of note, one same study could include different type of animals or genera, see the text for the total number of studies per genus), (c) total number of farms in the studies in which the given type of animals was studied (sometimes, the number of farms for the given type of animals was not explicitly mentioned or mentioned at all, hence the ratio between parentheses above the total number of farms: it informs about the number of articles where the number of farms was provided/the total number of articles where the given type of animals was studied, e.g. 1,801^(6/14)^ means that 1,801 farms were included in 6 studies/14, and that the number of farms was not explicitly mentioned or mentioned at all in 8 studies), (d) total number of farmers in the studies in which the given type of animals was studied (the ratio between parentheses above the total number of farmers informs about the number of articles where the number of farmers was provided/the total number of articles where the given type of animals was studied) and (e) total number of animals in the studies in which the given type of animals was studied (the ratio between parentheses above the total number of animals informs about the number of articles where the number of animals was provided/the total number of articles where the given type of animals was studied).

*Bos* was the most studied genus (n = 16 studies), followed by *Ovis* (n = 6), *Sus* (n = 2), *Capra* (n = 1), and *Gallus* (n = 1). Six studies out of 22 interested in two, or three genera/study. Breed was only specified in seven studies (32% of reviewed articles). The detailed numbers of farms, farmers, and animals per genus are presented in Figure 2 (when available).

### Farmer well-being assessment

Questionnaires used to assess farmer well-being differed considerably between quantitative studies (Table 3). Indeed, a total of 11 referenced questionnaires were used in seven studies (out of 16). Nine questionnaires out of 11 were validated (no explicit evidence of validation were retrieved for the Stanford Presenteeism Scale; Koopman *et al.* 2002, nor the Subjective well-being questionnaire from the Office for National Statistics of the United Kingdom in Crimes & Enticott 2019; Of note, the Psychosocial Index of Sonino & Fava 1998 compiles questions from different validated questionnaires). In other studies, questionnaires were either created (8 studies/16) or adapted (1 study/16). Different questionnaires could thus be used to assess one same aspect of a dimension. For instance, stress was assessed in six studies with five different questionnaires (Table 3). Only four referenced questionnaires were used in more than one study (the World Health Organisation Quality of Life Questionnaire [The WHOQOL Group 1998] in Fasina *et al.* 2010 and Nuvey *et al.* 2023; the Perceived Stress Scale [Cohen *et al.* 1983], Hospital Anxiety and Depression Scale [Zigmond & Snaith 1983], and refined version of the Connor-Davidson Resilience Scale [Campbell-Sills & Stein 2007] in King *et al.* 2021 and Medrano-Galarza *et al.* 2023). On average, 21 questions related to farmer well-being were answered per quantitative study (median = 6, IQR = [3; 22]).

**Table 3.** Synthesis of the methods used to assess the farmer well-being in the 16 reviewed studies with a quantitative approach, included in a scoping review aiming to map the methods used to describe — and compile pieces of evidence of — relationships between farmer well-being and animal welfare

Articles	Questionnaire	N_Items_^1^	Dimension	Aspect of the dimension	Indicator used^2^
Andreasen *et al.* (2020)	Farmer attitude questionnaire (adapted from Waiblinger *et al.* 2002)	1	Satisfaction	Work	Item score
Calderón-Amor *et al.* (2020)	Questionnaire made by Calderón-Amor *et al.* (2020)	1	Satisfaction	Work	Item score
Crimes and Enticott (2019)	Subjective well-being questionnaire from the Office for National Statistics of the United Kingdom (see Table 5 in the referred article)	4	Mental health Satisfaction Emotion	Stress, Anxiety Life Happiness	Total score of the questionnaire; Individual score of each item
	Warwick Edinburgh Mental Well-being Scale (Stewart-Brown and Janmohamed 2008)	7	Mental health Satisfaction Feeling	Worries Achievement, Social relationships Optimistic, Useful, Relaxed	Total score of the questionnaire; Individual score of each item
	Stanford Presenteeism Scale (Koopman *et al.* 2002)	6	Mental health Satisfaction Feeling	Worries Work Hopeless	Total score of the questionnaire; Individual score of each item
Fasina *et al.* (2010)	Psychosocial Index (Sonino & Fava 1998)	55	Mental health Physical and mental health Well-being	Stress, Distress Abnormal illness behaviour Overall	Four total scores: one for each aspect
	Symptom Questionnaire (Kellner 1987)	92	Mental health Physical health	Anxiety, Depression, Hostility-irritability Overall	Four total scores: one for each aspect
	World Health Organisation Quality of Life (WHOQOL) Questionnaire (The WHOQOL Group 1998)	26	Mental health Physical health Satisfaction	Overall Overall Social relationships, Environment	Four total scores: one for each aspect
Hansen and Østerås (2019)	Questionnaire made by Hansen and Østerås (2019)	7	Mental health Satisfaction Feeling	Stress, Worries Work, Future, Income, Social relationships Optimistic	Two synthetic factors: Farmer Occupational Well-being, and Farmer Stress (obtained after a factor analysis)
King *et al.* (2021)	Perceived Stress Scale (Cohen *et al.* 1983)	10	Mental health	Stress	Total score of the questionnaire
	Hospital Anxiety and Depression Scale (Zigmond & Snaith 1983)	14	Mental health	Anxiety, Depression	Two total scores: one for each aspect
	Refined version of the Connor-Davidson Resilience Scale (Campbell-Sills & Stein 2007)	10	Mental health	Resilience	Total score of the questionnaire
Lee *et al.* (2020)	Questionnaire made by Lee *et al.* (2020)	1	Mental health	Worries	Item score
Medrano-Galarza *et al.* (2023)	Perceived Stress Scale (Cohen *et al.* 1983)	10	Mental health	Stress	Total score of the questionnaire
	Hospital Anxiety and Depression Scale (Zigmond & Snaith 1983)	14	Mental health	Anxiety, Depression	Two total scores: one for each aspect
	Refined version of the Connor-Davidson Resilience Scale (Campbell-Sills & Stein 2007)	10	Mental health	Resilience	Total score of the questionnaire
Nuvey *et al.* (2023)	World Health Organisation Quality of Life (WHOQOL) Questionnaire (The WHOQOL Group 1998)	26	Mental health Physical health Satisfaction	Overall Overall Social relationships, Environment	Mean score of all items transformed to a scale from 0 to 100
Nuvey *et al.* (2020)	Short form of the Depression, Anxiety and Stress Scale (Henry & Crawford 2005; Lovibond & Lovibond 1995)	21	Mental health	Stress, Anxiety, Depression	Mean of the three sub-scores of each aspect
O’Kane *et al.* (2017)	Questionnaire made by O’Kane *et al.* (2017)	3	Emotion Feeling	Angry Frustrated, Miserable, Hopeless	Three scores: one for each item; Two components: one synthesizing negative emotion and feelings, one synthesising hopelessness (obtained after a principal component analysis)
Perrin *et al.* (2020)	Questionnaire made by Perrin *et al.* (2020)	4	Satisfaction	Work	Total score of the questionnaire
Pol *et al.* (2021)	Questionnaire made by Pol *et al.* (2021)	7	Satisfaction	Work	Three clusters of farms based on farmers’ satisfaction with their work, and the implemented management practices (obtained after a multiple correspondence analysis followed by an ascendant hierarchical clustering)
Rilanto *et al.* (2022)	Questionnaire made by Rilanto *et al.* (2022)	4	Satisfaction	Work	Four scores: one for each item; Three clusters of farmers based on farm manager’s attitude and opinions (obtained after a k-mean clustering algorithm)
Spigarelli *et al.* (2021)	Questions taken from the questionnaire of Fleury *et al.* (2008)	5	Satisfaction	Work, Social relationships, Environment, Future	Squared loading of the 5 variables (item scores) on the dimensions of 5 distinct principal component analyses
Vicic *et al.* (2022)	Questionnaire made by Vicic *et al*. (2022)	1	Well-being Mental health	General General	Item answer (Yes, No)

**1:** Number of items related to the assessment of farmer well-being in the questionnaire. **2:** Indicator of farmer well-being used in the articles to describe potential relationships between the farmer well-being and the welfare of their animals.

The most often studied farmer well-being dimension in the 16 quantitative studies was the satisfaction towards a life aspect (10 studies/16), followed by mental health (9/16), physical health (2/16), emotions (2/16), feelings (2/16), and overall well-being (2/16). On average, 1.7 dimensions were considered per study (median = 1.0; IQR = [1; 2]).

The most often studied aspect of dimensions in the 16 quantitative studies was the work satisfaction (8 studies/16), followed by psychological stress (5/16). On average, 3.7 aspects were considered per study (median = 3.5; IQR = [1; 4]).

The most used indicators to assess farmer well-being in quantitative studies were questionnaire scores (used in 12 studies/16), followed by factors, components or clusters synthesising information of several variables (5/16) (see Table 3; for more details regarding the construction of used indicators, see Supplementary material S1).

Semi-structured interviews followed by transcription and thematic analyses were carried out for most of the questionnaires in qualitative studies (5 studies/6; Table 4). Farmer well-being information was *a priori* approached at an overall level in interviews, allowing flexibility and freedom to farmers to share their experiences (five studies; Table 4). Methodological rational was explicitly mentioned in three studies. The derivation of theme being inductive or deductive was explicitly mentioned in one study.

**Table 4.** Synthesis of the methods used to assess the farmer well-being, animal welfare and their relationships in the six reviewed studies with a qualitative approach included in this scoping review (aiming to map the methods used to describe — and compile pieces of evidence of — relationships between farmer well-being and animal welfare)

Articles	Questionnaire	Methodological rationale (framework, model, philosophy)	Analysis	Derivation of themes	Indicator^**1**^
de Krom (2015)	Semi-structured interview about the management of group housing systems for gestating sows	Sociological practice theories developed especially since the 2000s. Stockpersons and sows conceptualised “as co-practitioners of farm-specific sow farming practices who enact and amend these practices by interactively performing and integrating its constitutive elements.” (p 422)	Transcription of interviews, followed by thematic analysis, with a focus “on the reasons why farmers chose a particular type of group housing barn interior layout and their references to the involvements of sows” (p 424)	Not explicitly mentioned	Verbatim representative of a relationship within an identified theme
Devitt *et al.* (2015)	Semi-structured interview about animal welfare incidents that occurred on farms	Narrative-based approach	Transcription of interviews, followed by thematic analysis	Not explicitly mentioned	Verbatim representative of a relationship within an identified theme
Noller *et al.* (2022)	Semi-structured in-depth interview about personal perspectives of farmers whom the farms were affected by *Mycoplasmopsis bovis*	Philosophical paradigm of pragmatism	Transcription of interviews, followed by a reflexive thematic analysis, guided by a multi-staged inductive approach (Braun and Clarke 2021)	Inductive	Verbatim representative of a relationship within an identified theme
Phythian and Glover (2019)	Semi-structured interview about perceptions and recollections of the first Schmallenberg disease outbreak on farmer well-being during the November-December 2012 lambing period	Not mentioned	Transcription of interviews, followed by thematic analysis	Not explicitly mentioned	Verbatim representative of a relationship within an identified theme
Vallance *et al.* (2023)	Semi-structured interview about impacts of facial eczema in ruminants on farmer well-being	Not mentioned	Transcription of interviews, followed by thematic analysis	Not explicitly mentioned	Verbatim representative of a relationship within an identified theme
van Calker *et al.* (2007)^2^	Farmer well-being: Physical load index (Hartman *et al*. 2005; 34 items) Animal welfare: TierGerechtheitsIndex–35L (TGI–35L; Bartussek, 1999; 24 items); Animal Health Index (Van Zeijts *et al.* 1999; 11 items used)	NA^3^	Modelling following the economic-environment LP-model (Berentsen and Giesen 1995)	NA	Four farm profiles presented in tables and obtained from models (*profile description based on 1) Farmer wellbeing indicator: index calculated by equally weighting the physical load index for back disorders and for neck, shoulder or upper extremities 2) Animal welfare indicator: relative scores of TGI–35L and Animal Health Index, grazing time, housing system*)

**1:** Indicator = Indicator showing a relationship between farmer well-being and the welfare of their animals. **2:** As a reminder, this study is based on a prospective longitudinal modelling with quantitative data. However, results are only descriptive, and no statistical correlation were described. Therefore, the descriptive results were used as if the study had a qualitative approach. **3:** Not Applicable (*cf note n°2*).

### Animal welfare assessment

Methods used to assess animal welfare differed between quantitative studies (Table 5). Most of questionnaires were created (8 studies/16) and referenced audits were only used in two studies (Welfare Quality® 2009: Andreasen *et al.* 2020 and Spigarelli *et al.* 2021; Protocol made by the EFSA Panel on Animal Health and Animal Welfare 2015). A new audit was created in one study (Hansen & Østerås 2019). In other studies, clinical observations based on protocol from previous studies (two studies: King *et al.* 2021; Medrano-Galarza *et al.* 2023), diagnostic tests (two studies: Fasina *et al.* 2010; Crimes & Enticott 2019), or a behavioural test (one study: Calderón-Amor *et al*. 2020) were carried out. On average, ten animal welfare indicators were measured per quantitative study (median = 2, IQR = [1; 9]).

**Table 5.** Synthesis of the methods used to assess animal welfare in the 16 reviewed studies with a quantitative approach included in this scoping review (aiming to map the methods used to describe — and compile pieces of evidence of — relationships between farmer well-being and animal welfare)

Articles	Questionnaire, test or audit	N_Items_^**1**^	Dimension	Aspect of the dimension	Indicator used^**2**^
Andreasen *et al.* (2020)	Welfare Quality® protocol for dairy cattle (Welfare Quality® project, 2009)	29	Nutrition Environment Health Behaviour Mental state Management	Overall, Feed, Water Overall, Free movement, Housing comfort, Thermal comfort Overall, Injuries Social Overall, Emotion Handling, Human-animal relationship, Pain management	One total score; Four principle scores (*Good feeding, Good housing, Good health and Appropriate behaviour*); Twelve criterion scores (*Absence of prolonged hunger, Absence of prolonged thirst, Comfort around resting, Thermal comfort, Ease of movement, Absence of injuries, Absence of disease, Absence of pain induced by management procedures, Expression of social behaviours, Expression of other behaviours, Good human-animal relationship, Positive emotional state*)
Calderón-Amor *et al.* (2020)	Escape test according to Bokkers *et al.* (2009)	1	Behaviour Mental state	Reactivity Attitude, Emotion	Escape score
Crimes and Enticott (2019)	Farm diagnostic regarding bovine tuberculosis	1	Health	Respiration	Farm status infection regarding bovine tuberculosis
Fasina *et al.* (2010)	Farm diagnostic regarding avian influenza H5N1	1	Health	Respiration	Farm infection status regarding avian influenza H5N1
Hansen and Østerås (2019)	Animal Welfare Indicator, based on a protocol made by Hansen and Østerås (2019)	47	Health Management	Body condition, Locomotion, Metabolism, Mortality, Reproduction, Udder health Longevity, Pain management	Total score of the Animal Welfare Indicator
King *et al.* (2021)	Clinical observations (body condition: Wildman *et al.* (1982); lameness: Flower and Weary (2006); Collection of milk quality data	3	Health	Body condition, Locomotion, Udder health	Prevalence of under-conditioned cows; Prevalence of over-conditioned cows; Prevalence of lameness; Prevalence of severe lameness; Somatic cell count
Lee *et al.* (2020)	Questionnaire made by Lee *et al.* (2020)	1	Health	Udder health	Farmer self-reported bulk tank somatic cell count
Medrano-Galarza *et al.* (2023)	Protocol of clinical observations (anaemia, body condition, cleanliness, diarrhoea, eye discharge, hoof growth, lameness, mastitis, respiration, skin lesions) and behavioural measures (temperament) (Dwyer *et al.* 2015)	11	Environment Health Mental state	Animal cleanliness Body condition, Digestion, Locomotion, Metabolic, Ocular, Udder health, Respiration, Skin lesions Calm	Prevalence of sheep with dirty fleece Clinical mastitis prevalence; Eye discharge prevalence; Hoof overgrowth prevalence; Lameness prevalence category; Prevalence of animals with skin lesions (Prevalence of respiratory problems; Prevalence of sheep with anaemia; Prevalence of sheep with poor body conditions; Prevalence of sheep with severe and extensive faecal soiling in the hindquarters Prevalence of ewes quiet during handling
Nuvey *et al.* (2023)	Questionnaire made by Nuvey *et al.* (2023)	2	Health	Mortality	Severity of herd mortality considering all causes of death; Severity of herd mortality due to diseases
Nuvey *et al.* (2020)	Questionnaire made by Nuvey *et al.* (2020)	2	Health	Mortality	Proportion of cattle lost over a one-year period
O’Kane *et al*. (2017)	Questionnaire used and presented in a fellow article (Winter *et al.* 2015)	10	Health Management	Locomotion Prevention and treatment of diseases	Presence of foot rot in the herd; Prevalence of lameness (both provided by farmers) Probability to be on a latent class [LC] according to the management practices implemented to treat or cull lame sheep (*LC1 = typical “best practices”; LC2 = “slow to act”; LC3 = “slow to act, delayed culling”; for more details, see O’Kane et al. (2017) result section 3.3*)
Perrin *et al.* (2020)	Questionnaire made by Perrin *et al.* (2020)	2	Environment	Outdoor access	Evolution of full time grazing between the conversion beginning into organic farming and the time of the survey (which occurred at least 6.5 to 7 years after the conversion beginning)
Pol *et al.* (2021)	Questionnaire made by Pol *et al.* (2021)	7	Environment Management	Housing enrichment, Housing type Animal handling, Pain management, Procedures, Socialization	Three clusters of farms based on farmers’ satisfaction with their work, and the implemented management practices (obtained after a multiple correspondence analysis followed by an ascendant hierarchical clustering)
Rilanto *et al.* (2022)	Questionnaire made by Rilanto *et al.* (2022)	2	Management	Culling	Culling rate; Mean age of culled cows; Culling rate as an active component in a k-mean clustering algorithm; Mean age of culled cows as an active component in a k-mean clustering algorithm
Spigarelli *et al.* (2021)	Protocol made by the European Food Security Authority (EFSA) Panel on Animal Health and Animal Welfare (2015)	18	Health	Body condition, Digestion, Lesions, Locomotion, Mortality, Reproduction, Respiration, Udder health	Overall animal welfare index as an active variable in a principal component analysis
	Qualitative Behaviour Assessment described in the Welfare Quality protocol for dairy cattle (Welfare Quality project, 2009)	20	Behaviour Mental state	Reactivity Attitude, Emotion	
Vicic *et al.* (2022)	Questionnaire made by Vicic *et al.* (2022)	1	Management	Euthanasia	Euthanasia of non-replacement dairy calves

**1:** Number of items related to the assessment of animal welfare in the questionnaire. **2:** Indicator of animal welfare used in the articles to describe potential relationships between the farmer well-being and the welfare of their animals.

The most often studied animal welfare dimension in the 16 quantitative studies was health (11 studies/16), followed by management (6/16), mental state (4/16), environment (4/16), behaviour (3/16), and nutrition (2/16). On average, 1.9 dimensions were considered per study (median = 1.5; IQR = [1; 2]).

The most often studied aspect of dimensions in the 16 quantitative studies were udder health and locomotion (both in 5 studies/16), closely followed by body condition, respiration, and mortality (each one in 4 studies/16). On average, 4.2 aspects were considered per study (median = 2.0; IQR = [1; 7]).

The most often used indicator to assess animal welfare was prevalence (of clinical observations or mortality) (6 studies/16), followed by total scores of animal welfare (3/16) (see Table 5; for more details regarding the construction of used indicators, see Supplementary material S1). No indicator was fully identical between studies, due to differences in indicator construction, or genus studied. For instance, the prevalence of under-conditioned animals was assessed with similar body condition scales ranging from 1 (emaciated) to 5 (obese) in both King *et al.* (2021) and Medrano-Galarza *et al.* (2023). However, this prevalence was calculated in different species (dairy cows and sheep, respectively), while using different low body condition score cut-offs (≤ 2.5 and < 2.0, respectively).

In qualitative studies, animal welfare indicators were mainly related to specific animal diseases (three studies) (Table 4). Besides, animal welfare indicators constituted a part of the interview context and were not measured nor identified from discussions with farmers (e.g. the occurrence of an animal welfare incident was a recruitment criterion in Devitt *et al.* 2015).

### Relationships description between farmer well-being and the welfare of their animals

Relationships between farmer well-being and the welfare of their animals were mostly explored with models in quantitative studies (9 studies/16) (Table 6). Most of the models were multivariable (n = 7 studies; with a manual backward [n = 4], forward [n = 2] or no [n = 1] stepwise process). No correction, for instance Bonferroni or Holm, was applied to the *P*-values in multivariable models. Association or comparison between only two variables were carried out less often (5/16; with tests like *t*-tests, Chi-squared, spearman rank correlation, or ANOVA). Component analysis and clustering were the least used statistical method to explore associations between farmer well-being and the welfare of their animals (3/16). Component analysis, as well as factor analysis, were however used to synthesise variables into components (or factors) and used thereafter as an output or explanatory variable in models (3/16) (Table 6).

**Table 6.** Synthesis of the statistics used to describe potential relationships between farmer well-being and the welfare of their animals in the 16 reviewed studies with a quantitative approach included in this scoping review

Articles	Statistical test	Statistical indicator	Data transformation
Andreasen *et al.* (2020)	Spearman Rank Correlation	Correlation coefficient	Answers to the Farmer attitude questionnaire synthesised into components with a Principal Component Analysis (*variables describing a component had to load ≥ 0.55, and not fulfil the loading criterion on any of the other components*)
Calderón-Amor *et al.* (2020)	Multivariable, mixed ordinal logistic regression model with a manual backward stepwise removal process *with P-value ≥ 0.05 as a cut-off to remove variables*), preceded by univariable, mixed ordinal logistic regression models with pen nested within farm as a random effect (*only variables with P-value ≤ 0.20 were offered to multivariable models*) Odds ratio	Estimated regression coefficient (*Low vs high satisfaction [high = referent])* Odds ratio (*Low vs high satisfaction [high = referent]*)	Categorisation of scores a) Work satisfaction: high (score 7) or low (score < 7) satisfaction; b) Escape scores: friendly calf (score 4), cautious calf (score 1, 2, or 3), and fearful calf (score 0)
Crimes and Enticott (2019)	Independent samples t-tests	Mean difference between farmers on farms having a restricted status regarding bovine tuberculosis *vs* free status	None
Fasina *et al.* (2010)	ANOVA	Mean difference between farmers on farms having experienced *vs* not experienced an avian influenza H5N1 outbreak	None
Hansen and Østerås (2019)	Structural equation model	Correlation coefficient	Factors obtained from farmer well-being items after a confirmatory factor analysis
King *et al.* (2021)	Multivariable mixed-effects linear regression models with a manual backward stepwise removal process (*with P-value > 0.10 as a cut-off to remove variables*), preceded by univariable regression models with sex as a covariate (*only variables with P-value < 0.25 were offered to multivariable models*)	Estimated regression coefficient	Natural logarithm applied to the percentage of over-conditioned cows; Natural logarithm applied to the (value +1) of the prevalence of severe lameness and under-conditioned cows since zeros were encountered
Lee *et al.* (2020)	Multivariable, mixed ordinal logistic regression model with a manual forward stepwise selection process (*with P-value < 0.05 as a cut-off to maintain variables*) Odds ratio	F-value Odds ratio	Cumulative logit link applied to item scores
Medrano-Galarza *et al.* (2023)	Multivariable mixed-effects linear regression models with a manual backward stepwise removal process (*with P-value > 0.05 as a cut-off to remove variables*), preceded by bivariate regression models (*only variables with P-value ≤ 0.20 were offered to multivariable models*)	Estimated regression coefficient	None
Nuvey *et al.* (2023)	Multivariable mixed-effects linear regression model without additional or removal process Univariable linear regression	Estimated regression coefficient Mean difference between farmers on farms with severe or low herd mortality	Herd mortality expressed in number of Tropical Livestock Unit [TLU] dead (cattle = 0.75 TLU, sheep and goat = 0.1 TLU) / total number of TLU of the herd
Nuvey *et al.* (2020)	Chi-squared test Multivariable linear regression model with a backward stepwise elimination process, using likelihood ratio tests, starting with the most plausibly complex model	Chi-squared Estimated regression coefficient	Categorisation of mental health scores: scores 1) above and 2) below the mean mental health score
O’Kane *et al.* (2017)	None, just description Multivariable multinomial logistic regression model with a forward stepwise selection process Multivariable negative binomial regression model with a forward stepwise selection process	Percentages Relative risk reduction of being in latent class “slow to act”, or “slow to act, delayed culling” regarding lameness Incidence Rate Ratio regarding period prevalence of lameness in sheep May 2012–April 2013	Profile of farmers towards lameness treatment obtained from answers about lameness treatment after latent class models; Component obtained from farmer well-being items after a Principal Component Analysis
Perrin *et al.* (2020)	Partial least square regression	Regression coefficient (R2)	None
Pol *et al.* (2021)	Multiple correspondence analysis followed by an ascendant hierarchical clustering	Cluster v-test values	None
Rilanto *et al.* (2022)	Spearman Rank Correlation K-mean clustering algorithm	Spearman correlation coefficient Average scores of variables at cluster-level	None
Spigarelli *et al.* (2021)	Principal component analysis	Squared loading on a dimension	Categorisation of satisfaction scores: unsatisfied = scores 1 and 2; neutral = score 3; satisfied = scores 4 and 5
Vicic *et al.* (2022)	Bayesian network model	Description of modality distribution (%)	None

**Figure 3.**
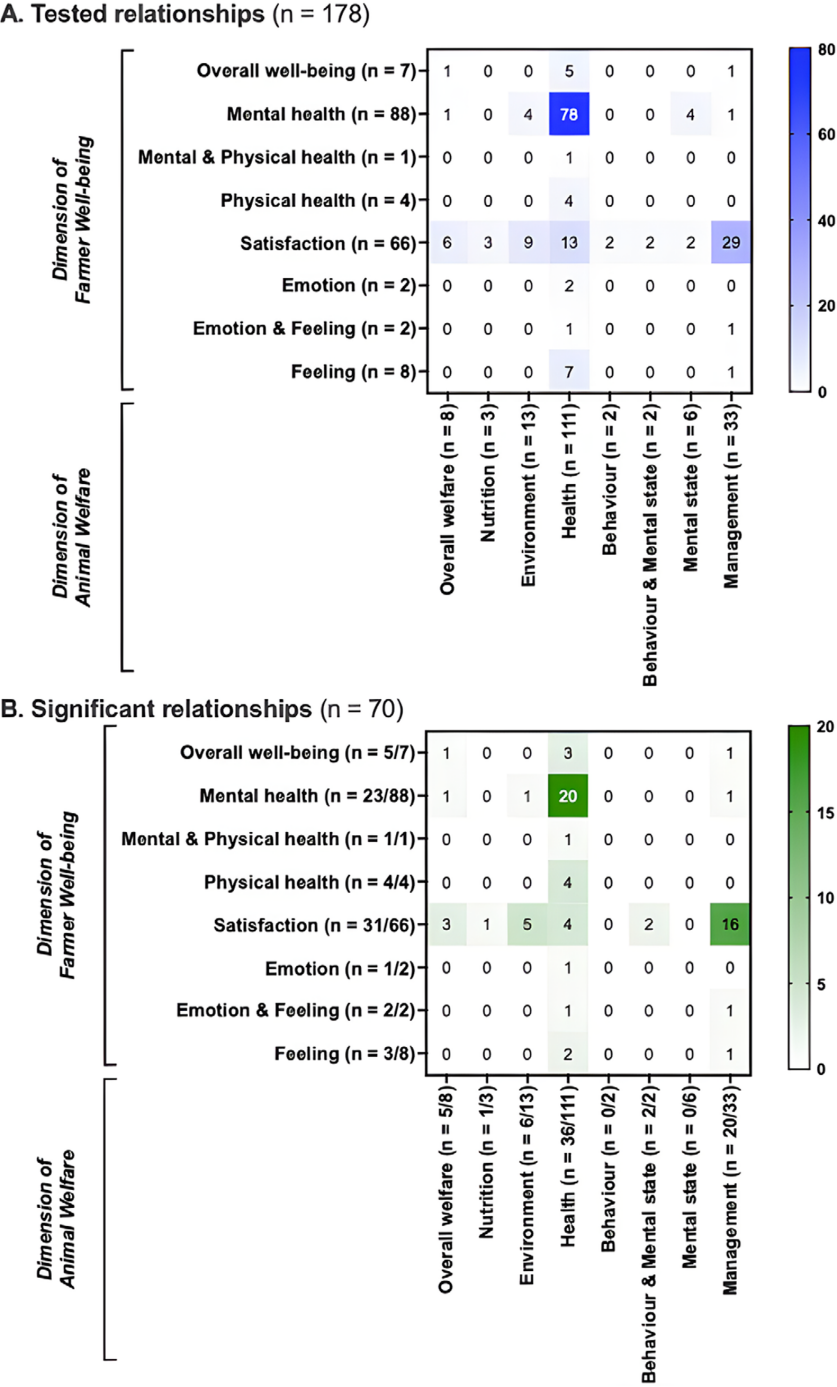
Heat maps at dimension-level of the distribution of the (a) 178 tested and (b) 70 significant relationships between indicators of farmer well-being and the welfare of their animals, as reported in the 16 reviewed quantitative studies included in this scoping review. Each cell shows the number of relationships tested between indicators corresponding to the two intersecting dimensions. For instance, ‘78’ in (a), second row and fourth column means that 78 relationships were tested between indicators of farmer mental health and of animal physical health; for (a) n = total number of tested relationships involving indicators from the given dimension and for (b) n = ratio of the number of significant relationships/the total number of tested relationships for the given dimension. Of note, a dimension in this article refers to distinct type of information regarding farmer well-being or animal welfare.

**Figure 4.**
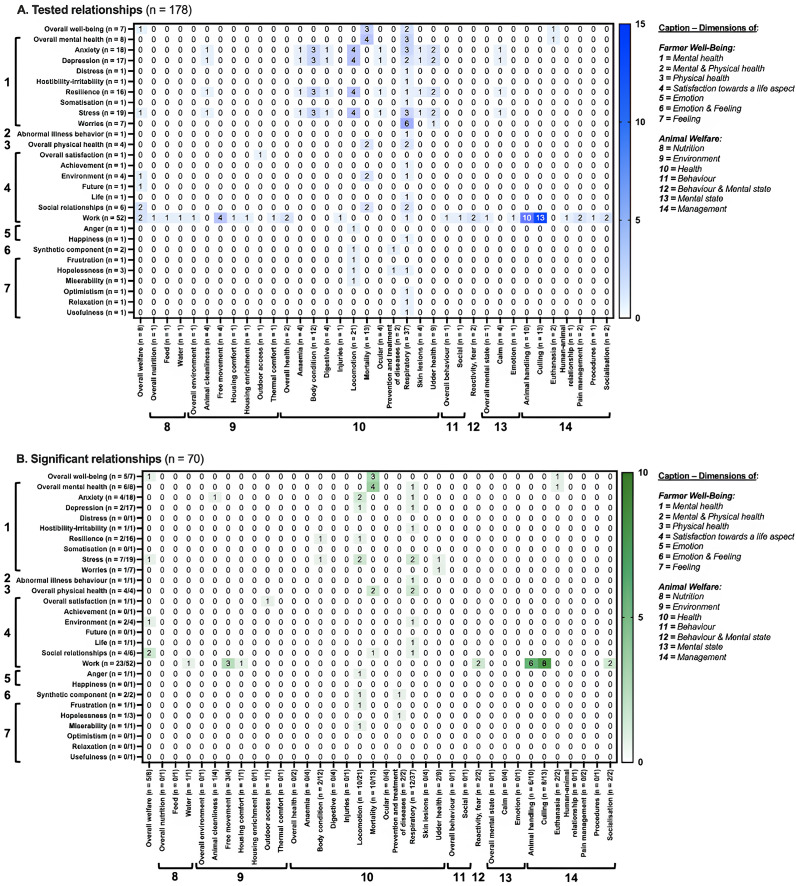
(a) Heat maps at aspect-level of the distribution of the (a) 178 tested relationships and (b) 70 significant relationships between indicators of farmer well-being and the welfare of their animals in the 16 reviewed quantitative studies included in this scoping review. For (a) n = total number of tested relationships concerning indicators from the given aspect, and for (b) n = ratio of the number of significant relationships/the total number of tested relationships for the given aspect. Of note, an aspect in this article refers to distinct type of information regarding a farmer well-being or animal welfare dimension.

At dimension-level, most of the 178 tested relationships concerned indicators of farmer mental health and of their animal physical health (n = 78 tested relationships), then indicators of satisfaction towards a farmer’s life aspect and the management of their animals (n = 29) (Figure 3[a]). At aspect-level, the most tested relationships concerned indicators of farmers’ work satisfaction and culling practices (n = 13), then animal handling (n = 10) (Figure 4[a]). Many relationships between dimensions are yet to be explored, such as that between farmer physical health and the environment of their animals.

### Pieces of evidence of the relationships between farmer well-being and the welfare of their animals

A total of 70 significant relationships were described in the 16 quantitative studies. At dimension-level in quantitative studies, the most frequent significant relationships concerned indicators of farmer mental health and the physical health of their animals (n = 20 significant relationships; i.e. 29% of the significant relationships in quantitative studies), then indicators of satisfaction towards a farmer’s life aspect and the management of their animals (n = 16; 23%) (Figure 3[b]). More precisely, at aspect-level, poorer overall farmer mental health (n = 4; 6%) and well-being (n = 3; 4%) were associated with higher herd mortality rates (Figure 4[b]). Also, a lower work satisfaction in farmers was associated with a higher culling rate and a younger age at culling (n = 8; 11%). A higher work satisfaction was associated with positive, calm and patient animal handling (n = 6; 9%).

**Figure 5.**
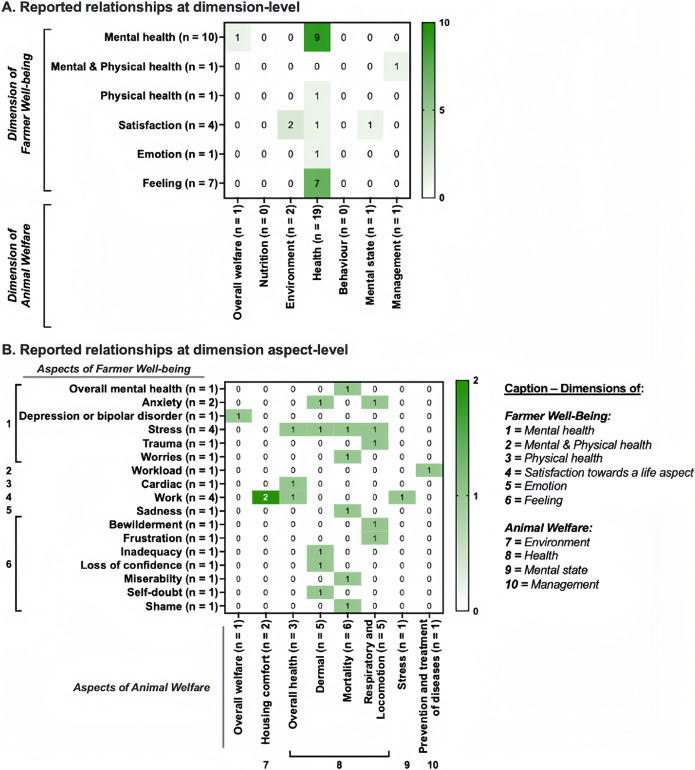
Heat maps of the distribution at (a) dimension-level and (b) aspect-level of 24 reported relationships between farmer well-being and the welfare of their animals in the six reviewed qualitative studies included in this scoping review (n = total number of reported relationships for the given dimensions or aspects. Of note, a dimension in this article refers to distinct type of information regarding farmer well-being or animal welfare, and an aspect refers to distinct type of information regarding a farmer well-being or animal welfare dimension).

A total of 24 reported relationships were reported in the six qualitative studies. At dimension-level in qualitative studies, the most frequent reported relationships concerned farmer mental health and the physical health of their animals (n = 9 reported relationships; i.e. 38% of the reported relationships in qualitative studies), then farmers’ feelings and the physical health of their animals (n = 7; 29%) (Figure 5[a]). More precisely, at aspect-level, animal mortality was reported to induce negative emotions and feelings (sadness, shame and miserability), increased stress and worries (n = 6; 25%) (Figure 5[b]).

Considering both quantitative and qualitative studies, 99% of pieces of evidence, from the overall to the aspect level, described that an improved farmer well-being was associated with an improved animal welfare, and *vice versa* (93 pieces out of 94; for more details regarding the pieces of evidence, see Supplementary material S2).

## Discussion

The results of this One Welfare scoping review are discussed below with two perspectives: (1) to provide a support for future study design, discussing observed gaps and limitations in methods used to date; and (2) to grasp the implications of compiled pieces of evidence on commercial farms.

### Methods of description

#### Description in the material and methods

Several reviewed studies lacked a complete description of the material and methods. First, sample sizes were not explicitly reported in more than half of the reviewed articles, especially for animals. Similarly, Winder *et al.* (2019), in their review which aimed at describing the completeness of reporting key items, found that animal sample size was not explicitly reported in 43% of 137 reviewed articles (of intervention trials published in the *Journal of Dairy Science* in 2017). Systematically reporting animal sample size would improve research reproducibility and help readers to better interpret the results. Moreover, providing a justification for a sample size would also strengthen methodological rigour. In the current review, farm, farmer and animal sample sizes were justified in only a minority of articles. Sample sizes could be justified in future studies considering the results and sample sizes of the reviewed studies. Second, the time of year in which data were collected was mentioned in only one-quarter of the reviewed articles. Yet, this information is relevant, as farmers’ physical and mental states can vary seasonally, partly due to workload increase related to crops from spring to summer and reduced daylight in winter (Westrin & Lam 2007; Ahmed *et al.* 2024). Seasonal effects could thus influence farmer well-being and subsequently affect the observed relationships with animal welfare outcomes. Reporting the study period is therefore important for contextualising results. Finally, animal breed was not reported in over two-thirds of the reviewed articles. Yet, breed could influence observed behaviours through differences in personality traits, which could in turn affect their associations with farmer well-being indicators (Morris *et al.* 1994; Sutherland *et al.* 2012; Setser *et al.* 2024). Moreover, breed could influence stress response indicators, as reported in Meishan and Large White pigs exposed to the same stressor (Désautés *et al.* 1997). Such differences could be partly explained by differences in densities of mineralocorticoid and glucocorticoid receptors, which are involved in the stress response (Perreau *et al.* 1999). To conclude, comprehensive reporting in the material and methods detailing the exact animal sample size, study period, breed specification, and more generally any relevant characteristics of the recruited sample would not only support interpretation of results but also promote reproducibility across studies.

#### Questionnaires used to assess farmer well-being

Different questionnaires were used to assess the same aspect of farmer well-being, but variations in item content can hinder comparability of results across quantitative studies. For instance, total score of psychological stress was measured using three different questionnaires across four of the reviewed studies (Perceived Stress Scale; Cohen *et al.* 1983; Psychosocial index, Sonino & Fava 1998; Short form of the Depression, Anxiety and Stress Scale, Henry & Crawford 2005). The three questionnaires differ in the numbers of items (14, 18 and 7, respectively), nature of the information assessed (feelings and thoughts, occurrence of stressors, or feelings and behaviours, respectively), and the time-frame considered (last month, last year, or last week, respectively). As a result, total stress scores from the three questionnaires are likely to reflect different aspects of stress, which may limit the comparability of their results. Limited comparability of results due to methodological variability is not specific to the reviewed topic, since similar issues were reported in other systematic reviews (*e.g.* regarding the diversity of refractometers used to assess bovine quality colostrum, or drugs administered for pain management in cattle castration) (Buczinski & Vandeweerd 2016; Nogues *et al.* 2025). Two suggestions could be considered to address the issue of results comparability in the reviewed topic. First, standardising the use of a single questionnaire for each well-being aspect would enable comparisons across studies. A limitation to standardisation could be its feasibility, considering the diversities of questionnaires and aspects related to farmer well-being. However, standardisation at a well-being aspect-level like psychological stress may be easier, since it targets a single specific construct. In that regard, standardisation should be conducted considering indicators of internal consistency and test-retest reliability, such as Cronbach’s alpha and intraclass correlation coefficient (ICC), respectively. Cronbach’s alpha between 0.7 and 0.9, and ICC ideally over 0.90 indicate satisfying reliability (Tavakol & Dennick 2011; Koo & Li 2016). Second, explicitly defining the investigated well-being aspect, rather than solely naming it, would provide a conceptual framework to guide meaningful comparison across studies (Ferraro *et al.* 2021). Such definitions would support both critical appraisal of the selected questionnaire and informed inclusion process in future meta-analysis. Future meta-analyses could, for instance, include only articles whose definition of the examined aspects align with their conceptual framework.

#### Synthesis of data into indicators

No animal welfare indicator was fully comparable across the reviewed quantitative studies. This outcome could be partly explained by the large number of welfare aspects assessed across a relatively small number of studies. However, even when indicators had the same name, differences in the unit scale of an explanatory variable in models (somatic cell count of tank: 1,000 cells mL^–1^ in King *et al.* 2021; 10,000 cells mL^–1^ in Lee *et al.* 2020), cut-off value to define a clinical sign (poor body conditions using the same scale range: ≤ 2.5 in King *et al.* 2021; < 2 in Medrano-Galarza *et al*. 2023), construction of total scores (total score of animal welfare: range = [0; 100] based on items from the six dimensions in Andreasen *et al.* 2020; range = [–141; 141] based on health and production items in Hansen & Østerås 2019), or studied genus hindered full comparability between the results of reviewed studies. Regarding total scores of animal welfare obtained from different audits, associations between scores could be tested to assess their comparability. However, as suggested by the results of Barry *et al.* 2024, such comparisons may be limited due to differences in audit and score constructions (out of the 240 correlations tested between [a] 10 sub-indicators of the Animal Welfare Indicator used in Hansen & Østerås 2019 and [b] 24 welfare measures of the Welfare Quality® used in Andreasen *et al.* 2020, 238 were either negligible or weak, and only two were moderate). To enhance comparability across studies, it is crucial to standardise welfare indicators whenever relevant, ensuring consistent measurement methods, unit scales, and thresholds where applicable.

Total score derived from farmer well-being questionnaires or animal welfare audits were used in three-quarters of the reviewed quantitative studies. Total score could reflect overall well-being (e.g. Nuvey *et al.* 2023) or welfare (e.g. Hansen & Østerås 2019), overall dimension (e.g. total score of mental health in Nuvey *et al.* 2023), or a single aspect (e.g. psychological stress in King *et al.* 2021). However, total score at a broader level than the aspects of a dimension could be overly synthetic to accurately describe relationships between farmer well-being and the welfare of their animals. As illustrated by the heat maps, farmer well-being and animal welfare encompass a wide range of dimensions and aspects. Aggregating different information into a total score could hinder a nuanced understanding of the relationships between farmer well-being and the welfare of their animals. For instance, in the studies by King *et al.* (2021) and Medrano-Galarza *et al.* (2023), the prevalence of severe lameness related to locomotion aspect of animal health was significantly associated with farmer psychological stress score in multivariable models, while the tank somatic cell count related to udder health aspect of animal health was not. Had both indicators been aggregated into a total welfare score, such differences in associations would likely have been masked. To accurately capture the relationships between farmer well-being and the welfare of their animals, a total score should be calculated at the aspect-level or lower, rather than at a dimension- or overall-level.

#### Data analysis

Modelling was the most frequently used method to describe associations between farmer well-being and animal welfare indicators in quantitative studies. Estimated regression and correlation coefficients provided insights into the direction and relative importance of an association. However, eta squared (η^2^) was not calculated in any of the reviewed studies. Eta squared is an effect size measure that quantifies the proportion of variance explained by each explanatory variable in a model (Cohen 1988). Including eta squared in model outputs would provide information complementary to *P*-values regarding the strength of an association. Various effect sizes can be measured according to the type of data and statistical test conducted (Fleiss 1994; Rosenthal 1994). The systematic reporting of effect size measures would enhance the understanding of the relationship importance between farmer well-being and the welfare of their animals.

The derivation of themes was not extensively described in qualitative studies. In most studies, it can remain unclear for readers unused to qualitative methodology whether the analytic process was an inductive, deductive or mixed approach. Such limited reporting raises concerns regarding transparency and interpretative depth, as the approach to theme derivation can strongly influence the findings (Braun & Clarke 2006). Future qualitative research exploring relationships between farmer well-being and the welfare of their animal would benefit from explicit description of the procedures used to derive themes (for instance, considering established reporting standards, such as ENTREQ; Tong *et al.* 2012).

#### Intersectoral collaborations

Intersectoral collaboration was carried out in only a minority of studies. In particular, no researcher in human sciences *a priori* took part in about two-thirds of the quantitative studies. It is worth noting that this proportion may be overestimated, since authors’ training was not checked. Some authors may have a background in human sciences and were only affiliated to department of animal sciences in the reviewed papers (or *vice versa*). In such cases, intersectoral collaborations would have been missed. Either way, intersectoral collaboration is strongly suggested since it could support the structuring and advancement of the One Welfare research, notably in the selection of appropriate questionnaires and indicators to assess farmer well-being. Even so, barriers to intersectoral research will have to be addressed to foster such collaborations. According to an umbrella review, two key barriers are the lack of shared vision between collaborators and insufficient funding dedicated to intersectoral research (Amri *et al.* 2022). Overcoming both barriers would require engaging communication between collaborators to identify common goals and creating funding grants dedicated to intersectoral research. Facilitating intersectoral research would not only benefit the One Welfare approach, but also the One Health approach, and contribute more broadly to addressing the 17 Sustainable Development Goals identified by the United Nations (United Nations 2015; Destoumieux-Garzón *et al.* 2018; Trowbridge *et al.* 2022).

### Pieces of evidence

A total of 94 pieces of evidence documenting relationships between farmer well-being and the welfare of their animals were compiled. Given their singularity, each piece of evidence could offer only a limited insight into a given aspect. Nonetheless, when considered collectively, pieces of evidence can provide synthetic and convergent results on a given aspect. For instance, the 16 pieces related to animal mortality consistently suggested that a poorer farmer well-being was related to the mortality of their animals, as an increased mortality was associated with poorer farmer overall well-being, overall mental health, and was reported to induce negative emotions, feelings, and more stress to farmers (Phythian & Glover 2019; Nuvey *et al.* 2020, 2023). To meaningfully appreciate the relationship between specific aspects of farmer well-being and animal welfare, it is therefore essential to consider the pieces of evidence collectively rather than individually. Of note, three factors should be accounted for when considering pieces of evidence collectively. It remains uncertain the extent to which differences in (1) the animal genera studied, (2) farm system within the same genus (e.g. conventional vs organic) and (3) industrialisation level of the country where the study was conducted might hinder the collective consideration of pieces of evidence. It is assumed that such collective consideration remains possible when the animal welfare indicator (or farmer well-being indicator) has (1) similar construction across studies and (2) comparable implications for farms across genera, farm systems, and industrialisation level (e.g. mortality, expressed as a percentage, can affect farm profitability regardless of animal genera, farm systems and industrialisation levels of countries). Besides, indicators too specific to a genus (or a farm system) may not exist in other genera (or farm systems), which would limit comparison *per se.*

From the overall to the aspect level, all pieces of evidence (except one) showed that an improved farmer well-being was associated with an improved animal welfare, and *vice versa.* This result could be pivotal in initiating a paradigm shift in how welfare improvement strategies are conceived on farms. Since pieces of evidence suggest that farmer well-being and animal welfare could be influenced by each other, on-farm strategies should not focus solely upon improving animal welfare but also improving farmer well-being. Indeed, farmers experiencing poor well-being in presence of poor animal welfare could need physical, social or psychological support. In extreme cases, incidents of poor animal welfare can even stem directly from severely deteriorated farmer well-being. As reported Devitt *et al.* (2015; pp 407–408): “*In one situation that involved a neglected animal illness, the farmer attributed the stress to marital and family separation, financial concerns and ill health: ‘When* [the separation] *started I got a heart attack and that sort of put the clatter on farming – the pressure of it all was too much, the stress, and I just couldn’t cope with it’*”. Pieces of evidence thus also suggest to not overwhelm farmers. This suggestion is particularly important given public expectation towards animal welfare improvement (Clark *et al.* 2016; Alonso *et al.* 2020). Farmers can indeed be held accountable by the public for what can be perceived as poor animal welfare management (Duley *et al.* 2022; Kuenzler *et al.* 2024). Yet, this accountability can be stressful for farmers (Kallioniemi *et al.* 2016). A One Welfare perception from public, acknowledging the close interconnections between farmer well-being and the welfare of their animals, could help foster a more constructive and empathetic approach to improving farm animal welfare.

### Further research

Methods used to assess farmer well-being in future studies could differ from those currently employed. First, a greater number of study designs could be longitudinal. While most of the reviewed studies were cross-sectional, longitudinal studies could provide complementary insights into how farmer well-being and the welfare of their animals co-evolve over time. Two retrospective longitudinal studies from the same cohort, published after the final literature search, showed such results (Steen *et al.* 2024, 2025). For instance, over a two-year period, an overall relative livestock welfare score had a significant greater decrease on farms where farmers experienced symptoms of anxiety, depression, psychological distress, or poor life satisfaction, compared to farms where the farmer did not experience such symptoms (Steen *et al.* 2024; for more detail regarding the study design, see their companion-paper: Torske *et al.* 2024). Besides, longitudinal studies could allow researchers to establish causal relationships, thereby providing a higher level of evidence than cross-sectional associations described to date (Altman & Krzywinski 2015; Lee *et al.* 2024). Identifying causal relationships could help to develop a One Welfare monitoring on commercial farms and enable the development of early intervention strategies, based on the evolution of key indicators. For instance, deteriorated animal welfare indicators could serve as a warning sign of deteriorating farmer well-being. In such cases, sentinels like veterinarians or other farm advisors, could report this information to farm social workers to prompt timely support. Nonetheless, such a system still needs to be developed and strengthened across countries. On one hand, sentinels must be trained to identify distress in farmers and to hand over cases to an appropriate support service, while maintaining emotional boundaries to protect themselves (Gunn & Hughes-Barton 2022; Wheeler *et al.* 2025). On the other hand, the physical and psychosocial support landscape that takes over sentinels needs to be reinforced in many countries, as a farm social worker or accidental counsellors are neither available nor trained everywhere (Gunn & Hughes-Barton 2022). Second, complementary questionnaires or indicators to those used to date could be employed to expand the scope of investigated aspects. For instance, using a referenced questionnaire to assess farmers’ satisfaction towards life aspects which has not been done in the reviewed studies could be considered (see for instance the Personal Wellbeing Index – Adult version, International Wellbeing Group 2024).

Aspects of both farmer well-being and animal welfare could be described more specifically, frequently and across a broader scope. Regarding farmer well-being, aspects of physical health could be described more specifically, since only overall physical health was considered in reviewed studies (Fasina *et al.* 2010; Nuvey *et al.* 2023). Feelings and emotions could be assessed more frequently, since they were described in only a limited number of quantitative associations (12 in total) and qualitative reports (8 in total) (O’Kane *et al.* 2017; Phythian & Glover 2019; Vallance *et al.* 2023). Besides, farmer burn-out was not considered at all, despite an average prevalence of 13.7% reported in the systematic review of O’Shaughnessy *et al.* (2022) (range = [9.0; 19.0]; average prevalence based on four studies carried out in Canada, Finland, Morocco and New Zealand, and a cumulative sample size of 1,904 farmers). As for animal welfare, the scope of behaviour and mental state aspects could be expanded, as only two and four indicators were used, respectively (Andreasen *et al.* 2020; Calderón-Amor *et al.* 2020; Medrano-Galarza *et al.* 2023). For instance, long-term stress, an aspect of mental state, can be assessed through concentrations of hair cortisol in mammals (Heimbürge *et al.* 2019), or feather corticosterone in poultry (Romero & Fairhurst 2016). Enhancing the specificity, frequency, and breadth of the aspects assessed would allow for a more comprehensive examination of the relationships between farmer well-being and the welfare of their animal. Of note, broadening the scope of assessed aspects should not be detrimental to cross-study comparability. Future studies could thus rely upon previously used methods or integrate only complementary ones.

Further research is needed to deepen our understanding of the relationships between farmer well-being and the welfare of their animals. First, while existing pieces of evidence show converging patterns, each piece is almost unique and requires confirmation. Second, numerous potential relationships have yet to be explored. The heat maps considering relationships retrieved from quantitative and qualitative studies help identity gaps that had not been explored as of the literature search (September 3^rd^, 2024). However, hypotheses should be formulated prior to future exploration, to justify the relevance of examining certain relationships and to further support the One Welfare approach. For instance, relationships between animal housing comfort and farmer physical health have not been explored. Still, musculoskeletal disorder prevalence is high in farming populations, which could be detrimental to routine tasks involving physical force, like bedding management (prevalence of musculoskeletal pain or discomfort affecting work of 3,288 US mid-western farmers in 2020: 65.4%; prevalence of musculoskeletal pain ≥ 3 months of 1,143 Norwegian farmers between 2017 and 2019: 52.1%) (Chengane *et al.* 2021; Steen *et al.* 2023). Third, relationships between farmer well-being and the welfare of their animals have undergone greater scrutiny in certain genera (*e.g. Bos*) compared to others (e.g. *Sus*, *Gallus*, or *Capra*). Further research on under-studied genera would deepen our knowledge and may enable the adaption of different One Welfare approaches across animal genera. Fourth, exploring relationships between farmer well-being and the welfare of their animals across diverse farming systems is of interest, given that most reviewed studies focused upon conventional farm systems in industrialised countries. Such exploration may also enable the adaption of different One Welfare approaches across systems and world regions. Finally, future qualitative research could provide complementary findings to quantitative studies and allow generation of future hypotheses. For instance, farmers’ opinions on the relationships between their well-being and the welfare of their animals could be explored.

### Study limitations

Production indicators were not eligible as welfare indicators in this review. Still, production indicators could fall within the welfare definition used (Anses 2018). Moreover, the scientific conception of welfare proposed by Fraser *et al.* (1997) includes the notion of “*growth and normal functioning of physiological*” system, which can, in part, be measured by production indicators. Had production indicators been eligible, additional articles could have met the inclusion criteria, potentially increasing the number of pieces of evidence retrieved (see, for instance, Hanna *et al.* 2009). However, a production indicator remains a non-specific proxy-outcome for welfare. Production indicator, like milk yield, can be high despite a degraded health status, and thus not necessarily reflect a positive welfare state (Coignard *et al.* 2014). Moreover, production indicators are related to farm profitability (Krpálková *et al.* 2014; Schorr & Lips 2018). It would have been difficult to discern whether any association with farmer well-being indicator stemmed from improved animal welfare or economic performance. Given this scoping review aimed to provide, for the first time, a map as robust as possible of the relationships between farmer well-being and the welfare of their animals, it was decided to limit eligible indicators to those directly reflecting animal’s state, or management practices likely to influence their quality of life.

Publication bias may explain why 93 out of 94 pieces of evidence were positive relationships. Negative relationships may be less likely to be published in journals (DeVito & Goldacre 2019). Publishing such results would nonetheless be useful to provide a better understanding of the relationships between farmer well-being and the welfare of their animals. It would subsequently help to tailor the One Welfare approach to different farm contexts, considering potentially different profiles of relationships between farmer well-being and the welfare of their animals.

Sample sizes in reviewed quantitative studies were often small and rarely justified. Small samples in quantitative studies can introduce biases that may limit the validity of results. Selection or volunteer biases may have occurred, which would hinder sample representativeness (Brassey *et al.* 2017; Nunan *et al.* 2017). When representativeness is not ensured, results cannot be extrapolated. Indeed, the presence and importance of some relationships may have been inaccurately described due to a random error. However, it is worth noting that the One Welfare approach remains relatively recent (Pinillos *et al.* 2016), and some study designs have been described as exploratory (King *et al.* 2021). Results based on small samples in quantitative studies should therefore be interpretated as exploratory rather than generalisable. This does not diminish their importance, as they provide a first overview of the relationships between farmer well-being and the welfare of their animals, and are useful for generating further hypotheses. In contrast, small sample sizes in qualitative studies are not a limitation, considering the saturation principle (Saunders *et al.* 2018). Moreover, some sampling techniques as the maximum variation sampling used in Noller *et al.* (2022) enable the collection of a wide diversity of data with a small sample (since interviewees with distinct experiences, or perspectives on a topic are recruited).

A scoping review does not provide a critical appraisal of individual sources of evidence (Munn *et al.* 2022). Therefore, neither the quality of the data collected nor the particular risk of bias in included studies were assessed. Some studies may have not been included with a critical appraisal, like that of Lee *et al.* (2020). The animal welfare indicator used in their study was a bulk tank somatic cell count self-reported by dairy farmers. The use of self-reported data can limit the accuracy and validity of the results in that particular study. Nonetheless, such a limitation does not compromise the validity of the results in this scoping review. Rather, the results presented here should be considered as a first step in mapping the existing evidence on the relationships between farmer well-being and the welfare of their animals.

### Animal welfare implications

An improved animal welfare was associated with an improved farmer well-being in 93 pieces of evidence out the 94 retrieved. This suggests that welfare improvement strategies on farms should not solely focus upon improving animal welfare, but also farmer well-being. An improved farmer well-being may indeed be a facilitator to reach and sustain an improved animal welfare.

## Conclusion

This One Welfare scoping review contributed to mapping the description of the relationships between farmer well-being and the welfare of their animals. The results showed the need to standardise the methods used to describe relationships, in order to improve cross-study comparison. The comparability across studies is essential for building a coherent body of evidence and increase its strength over time. Suggestions were proposed in the discussion to address comparability issues, gaps and limitations identified in the methods used to date. Importantly, the scoping review compiled for the first time pieces of evidence regarding the relationships between farmer well-being and the welfare of their animals. All identified pieces of evidence (except one) showed that improved farmer well-being was associated with improved welfare of their animals, and *vice versa.* This result suggests that strategies to improve welfare on farms should not focus solely on animals, but also on farmer well-being. Further research is needed to generate additional pieces of evidence to strengthen the importance of the One Welfare approach on commercial farms. In particular, a deeper exploration of causal relationships could help identify early warning indicators of deterioration in both farmer well-being and animal welfare, thereby enabling timely interventions.

## Supporting information

10.1017/awf.2025.10056.sm001 Levallois et al. supplementary material 1 Levallois et al. supplementary material

10.1017/awf.2025.10056.sm002 Levallois et al. supplementary material 2 Levallois et al. supplementary material

## Supplementary material

The supplementary material for this article can be found at http://doi.org/10.1017/awf.2025.10056.
