# Supplementary material for: Relationships between farmer well-being and the welfare of their animals: A One Welfare scoping review
Source: Anim Welf. 2026 Jan 15;35:e3. doi: 10.1017/awf.2025.10056 (PMC12817228; doi:10.1017/awf.2025.10056)
Supplement: Levallois et al. supplementary material 2 — Levallois et al. supplementary material [file S0962728625100560sup002.pdf]

Supplementary Material S1 – Tables 3 and 5 with more details regarding the construction of used indicators to respectively assess farmer well-being and animal welfare in the 16 reviewed studies with a quantitative approach included in the following scoping review: "*Relationships between farmer well-being and the welfare of their animals: a One Welfare scoping review*"

Pierre Levallois[1,2], Sébastien Buczinski[1], Marion Desmarchelier[1], Sonia Lupien[2,3], Marianne Villettaz-Robichaud[1]

[1] Department of Clinical Sciences, Faculty of Veterinary Medicine, University of Montreal, 3200 rue Sicotte, St-Hyacinthe, QC, J2S 2M2, Canada

[2] Centre for Studies on Human Stress, Research Center of the Montreal Mental Health University Institute, 7331 rue Hochelaga, Montréal, QC, H1N 3V2, Canada

[3] Department of Psychiatry, University of Montreal, 6128 succursale Centre-ville, Montréal, QC, H3C 3J7, Canada

Corresponding authors: pierre.levallois@umontreal.ca; marianne.villettaz.robichaud@umontreal.ca

Table 3: Synthesis of the methods used to assess the farmer well-being in the 16 reviewed studies with a quantitative approach included in a scoping review (aiming to map the methods used to describe —and compile pieces of evidence of— relationships between farmer well-being and animal welfare) (detailed version; part 1/2)

**Articles**	**Questionnaire**	**N_{Items}[1]**	**Dimension**	**Aspect of the dimension**	**Indicator used[2]**
Andreasen *et al.* (2020)	Farmer attitude questionnaire (adapted from Waiblinger *et al.* (2002))	1	Satisfaction	Work	Item score (1 = Strongly agree, 7 = Strongly disagree)
Calderón-Amor *et al.* (2020)	Questionnaire made by Calderón-Amor *et al. (2020)*	1	Satisfaction	Work	Item score (1 = Not at all, 7 = A lot)
Crimes and Enticott (2019)	Subjective well-being questionnaire from the Office for National Statistics of the United Kingdom (see Table 5 in the referred article)	4	Mental health<br>Satisfaction<br>Emotion	Stress, Anxiety<br>Life<br>Happiness	Total score of the questionnaire (0-40); Individual score of each item (0 = Not at all, 10 = Completely)
	Warwick Edinburgh Mental Well-being Scale (Stewart-Brown and Janmohamed 2008)	7	Mental Health<br>Satisfaction<br>Feeling	Worries<br>Achievement, Social relationships<br>Optimistic, Useful, Relaxed	Total score of the questionnaire (7-35); Individual score of each item (1 = Strongly disagree, 5 = Strongly agree)
	Stanford Presenteeism Scale (Koopman *et al.* 2002)	6	Mental health<br>Satisfaction<br>Feeling	Worries<br>Work<br>Hopeless	Total score of the questionnaire (6-30); Individual score of each item (1 = Strongly disagree, 5 = Strongly agree)
Fasina *et al.* (2010)	Psychosocial Index (Sonino and Fava 1998)	55	Mental health<br>Physical and mental health<br>Well-being	Stress, Distress<br>Abnormal illness behaviour<br><br>Overall	Four total scores: one for each aspect (stress: 0-18, distress: 0-45, abnormal illness behaviour: 0-9, where a higher score indicates a poorer well-being state; overall well-being: 0-23, where a higher score indicates a better well-being state)
	Symptom Questionnaire (Kellner 1987)	92	Mental health<br>Physical health	Anxiety, Depression, Hostility-irritability<br>Overall	Four total scores: one for each aspect (0-17, where a higher score indicates a poorer well-being state)
	World Health Organization Quality of Life (WHOQOL) Questionnaire (The WHOQOL Group 1998)	26	Mental health<br>Physical health<br>Satisfaction	Overall<br>Overall<br>Social relationships, Environment	Four total scores: one for each aspect (4-20, where a higher score indicates a better well-being state)
Hansen and Østerås (2019)	Questionnaire made by Hansen and Østerås (2019)	7	Mental health<br>Satisfaction<br>Feeling	Stress, Worries<br>Work, Future, Income, Social relationships<br>Optimistic	Two synthetic factors: Farmer Occupational Well-being, and Farmer Stress (obtained after a factor analysis)
King *et al.* (2021)	Perceived Stress Scale (Cohen *et al.* 1983)	10	Mental health	Stress	Total score of the questionnaire (0-40, where a higher score indicates a higher level of perceived stress)
	Hospital Anxiety and Depression Scale (Zigmond and Snaith 1983)	14	Mental health	Anxiety, Depression	Two total scores: one for each aspect (0-21, where a higher score indicates a higher level of anxiety or depression)
	Refined version of the Connor-Davidson Resilience Scale (Campbell-Sills and Stein 2007)	10	Mental health	Resilience	Total score of the questionnaire (0-40, where a higher score indicates a greater resilience)
Lee *et al.* (2020)	Questionnaire made by Lee *et al.* (2020)	1	Mental health	Worries	Item score (1 = Strongly disagree, 7 = Strongly agree)

1: Number of items related to the assessment of farmer well-being in the questionnaire. **2:** Indicator of farmer well-being used in the articles to describe a potential relationship between the farmer well-being and the welfare of their animals.

Table 3: Synthesis of the methods used to assess the farmer well-being in the 16 reviewed studies with a quantitative approach included in a scoping review (aiming to map the methods used to describe —and compile pieces of evidence of— relationships between farmer well-being and animal welfare) (detailed version; part 2/2)

Articles	Questionnaire	N_{Items}[1]	Dimension	Aspect of the dimension	Indicator used[2]
Medrano-Galarza *et al.* (2023)	Perceived Stress Scale (Cohen *et al.* 1983)	10	Mental health	Stress	Total score of the questionnaire (0-40, where a higher score indicates a higher level of perceived stress)
	Hospital Anxiety and Depression Scale (Zigmond and Snaith 1983)	14	Mental health	Anxiety, Depression	Two total scores: one for each aspect (0-21, where a higher score indicates a higher level of anxiety or depression)
	Refined version of the Connor-Davidson Resilience Scale (Campbell-Sills and Stein 2007)	10	Mental health	Resilience	Total score of the questionnaire (0-40, where a higher score indicates a greater resilience)
Nuvey *et al.* (2023)	World Health Organization Quality of Life (WHOQOL) Questionnaire (The WHOQOL Group 1998)	26	Mental health<br>Physical health<br>Satisfaction	Overall<br>Overall<br>Social relationships, Environment	Mean score of all items (1-5) multiplied by four, then transformed to a scale from 0 to 100 (where a higher score indicates a better well-being state); Mean score of each aspect (1-5) multiplied by four, then transformed to a scale from 0 to 100 (100 being a higher well-being state)
Nuvey *et al.* (2020)	Short form of the Depression, Anxiety and Stress Scale (Henry and Crawford 2005; Lovibond and Lovibond 1995)	21	Mental health	Stress, Anxiety, Depression	Mean of the three sub-scores of each aspect (0-21, where a higher score indicates a poorer mental health state). Each sub-score was obtained summing item scores (0-3, 0 = non-experience of the negative emotion, 3 = frequent negative emotion experience)
O'Kane *et al.* (2017)	Questionnaire made by O'Kane *et al.* (2017)	3	Emotion<br>Feeling	Angry<br>Frustrated, Miserable, Hopeless	Three scores: one for each item (1 = Strongly disagree, 5 = Strongly agree); Two components: one synthesizing negative emotion and feelings, one synthesizing hopelessness (obtained after a principal component analysis)
Perrin *et al.* (2020)	Questionnaire made by Perrin *et al.* (2020)	4	Satisfaction	Work	Total score of the questionnaire (4-16, where a higher score indicates a higher satisfaction level)
Pol *et al.* (2021)	Questionnaire made by Pol *et al.* (2021)	7	Satisfaction	Work	Three clusters of farms based on farmers' satisfaction with their work, and the implemented management practices (obtained after a multiple correspondence analysis followed by an ascendant hierarchical clustering)
Rilanto *et al.* (2022)	Questionnaire made by Rilanto *et al.* (2022)	4	Satisfaction	Work	Four scores: one for each item (1 = Completely disagree, 7 = Completely agree); Three clusters of farmers based on farm manager's attitude and opinions (obtained after a k-mean clustering algorithm)
Spigarelli *et al.* (2021)	Questions taken from the questionnaire of Fleury *et al.* (2008)	5	Satisfaction	Work, Social relationships, Environment, Future	Squared loading of the 5 variables (item scores) on the dimensions of 5 distinct principal component analyses
Vicic *et al.* (2022)	Questionnaire made by Vicic *et al.* (2022)	1	Well-being<br>Mental health	General<br>General	Item answer (Yes, No)

1: Number of items related to the assessment of farmer well-being in the questionnaire. **2:** Indicator of farmer well-being used in the articles to describe potential relationships between the farmer well-being and the welfare of their animals.

Table 5: Synthesis of the methods used to assess animal welfare in the 16 reviewed studies with a quantitative approach included in a scoping review (aiming to map the methods used to describe —and compile pieces of evidence of— relationships between farmer well-being and animal welfare) (detailed version; part 1/3)

Articles	Questionnaire, test or audit	N_{Items}[1]	Dimension	Aspect of the dimension	Indicator used[2]
Andreasen *et al.* (2020)	Welfare Quality protocol for dairy cattle (Welfare Quality project, 2009)	29	Nutrition<br>Environment<br><br>Health<br>Behaviour<br>Mental State<br>Management	Overall, Feed, Water<br>Overall, Free movement, Housing comfort, Thermal comfort<br>Overall, Injuries<br>Social<br>Overall, Emotion<br>Handling, Human-animal relationship, Pain management	One total score; Four principle scores (*Good feeding, Good housing, Good health and Appropriate behaviour*); Twelve criterion scores (*Absence of prolonged hunger, Absence of prolonged thirst, Comfort around resting, Thermal comfort, Ease of movement, Absence of injuries, Absence of disease, Absence of pain induced by management procedures, Expression of social behaviours, Expression of other behaviours, Good human-animal relationship, Positive emotional state*) (0-100 for each score, where a higher score indicates a higher animal welfare level)
Calderón-Amor *et al.* (2020)	Escape test according to *Bokkers et al.* (2009)	1	Behaviour<br>Mental State	Reactivity<br>Attitude, Emotion	Escape score (0 = Animal avoided eye contact, 4 = Animal could be touched)
Crimes and Enticott (2019)	Farm diagnostic regarding bovine tuberculosis	1	Health	Respiration	Farm status infection regarding bovine tuberculosis (Free, Under restrictions)
Fasina *et al.* (2010)	Farm diagnostic regarding avian influenza H5N1	1	Health	Respiration	Farm infection status regarding avian influenza H5N1 (Positive, Negative; based on two laboratory tests)
Hansen and Østerås (2019)	Animal Welfare Indicator, based on a protocol made by Hansen and Østerås (2019)	47	Health<br><br><br>Management	Body condition, Locomotion, Metabolism, Mortality, Reproduction, Udder health<br>Longevity, Pain management	Total score of the Animal Welfare Indicator (-141, 141, where a higher score indicates a higher animal welfare level)
King *et al.* (2021)	Clinical observations (body condition: Wildman *et al.* (1982); lameness: Flower and Weary (2006); Collection of milk quality data	3	Health	Body condition, Locomotion, Udder health	Prevalence of under-conditioned cows (*i.e.* body condition score ≤ 2.5; 1 = emaciated, 5 = obese); Prevalence of over-conditioned cows (*i.e.* body condition score ≥ 3.5); Prevalence of lameness (*i.e.* lameness score ≥ 3; 1 = movement easy and fluid, 5 = limited ability to move); Prevalence of severe lameness (*i.e.* lameness score ≥4); Somatic cell count (x 1,000 cells / mL);
Lee *et al.* (2020)	Questionnaire made by Lee et al. (2020)	1	Health	Udder health	Farmer self-reported bulk tank somatic cell count (x 10,000 cells / mL)

1: Number of items related to the assessment of animal welfare in the questionnaire. **2:** Indicator of animal welfare used in the articles to describe potential relationships between the farmer well-being and the welfare of their animals.

Table 5: Synthesis of the methods used to assess animal welfare in the 16 reviewed studies with a quantitative approach included in a scoping review (aiming to map the methods used to describe —and compile pieces of evidence of— relationships between farmer well-being and animal welfare) (detailed version; part 2/3)

Articles	Questionnaire, test or protocol	N_{Items}[1]	Dimension	Aspect of the dimension	Indicator used[2]
Medrano-Galarza *et al.* (2023)	Protocol of clinical observations (anaemia, body condition, cleanliness, diarrhoea, eye discharge, hoof growth, lameness, mastitis, respiration, skin lesions) and behavioural measures (temperament) (Dwyer *et al.* 2015)	11	Environment	Animal cleanliness	Prevalence of sheep with dirty fleece (*i.e.* dirtiness score > 1; 1 = clean and dry, 3 = very wet and dirty)
			Health	Body condition, Digestion, Locomotion, Metabolic, Ocular, Udder health, Respiration, Skin lesions	Clinical mastitis prevalence (Presence defined by individual observations and palpations of the udder for redness and changes in appearance); Eye discharge prevalence (0 = Absence, 1 = Presence); Hoof overgrowth prevalence (0 = Absence, 1 = Presence); Lameness prevalence category (dichotomized as high: > 5% vs low: ≤ 5%; 0 = No lameness, 2 = Severe lameness); Prevalence of animals with skin lesions (0 = Absence of skin lesion on every body parts, 1 = Evidence of at least one skin lesion); Prevalence of respiratory problems (Presence defined by individual observations of breath sound, coughing and nasal discharge); Prevalence of sheep with anaemia (*i.e.* conjunctiva score ≥ 4; 1 = Non-anaemic animal (red conjunctiva), 5 = Severely anaemic sheep (white conjunctiva)); Prevalence of sheep with poor body conditions (*i.e.* body condition score < 2; 1 = emaciated, 5 = obese); Sheep with severe and extensive faecal soiling in the hindquarters (*i.e.* faecal score > 3; 1 = No faecal matter present, 5 = Dirt and accumulation of faecal matter extended down to the legs and hock)
			Mental state	Calm	Prevalence of ewes quiet during handling (temperament assessed when animals were released after clinical observations; Temperament = calm, alert uncomfortable, or depressed);
Nuvey *et al.* (2023)	Questionnaire made by Nuvey *et al.* (2023)	2	Health	Mortality	Severity of herd mortality considering all causes of death (low: < 1%; 1% ≤ moderate < 31%; severe: ≥31%; based on quartiles); Severity of herd mortality due to diseases (low: < 1%; 1% ≤ moderate < 18%; severe: ≥18%; based on quartiles)
Nuvey *et al.* (2020)	Questionnaire made by Nuvey *et al.* (2020)	2	Health<br>Other	Mortality<br>Other (theft of cattle)	Proportion of cattle lost over a one-year period (*for several causes: diseases, theft, or also pasture shortage*)
O'Kane *et al.* (2017)	Questionnaire used and presented in a fellow article (Winter *et al.* 2015)	10	Health	Locomotion	Presence of foot rot in the herd; Prevalence of lameness (both provided by farmers)
			Management	Prevention and treatment of diseases	Probability to be on a latent class [LC] according to the management practices implemented to treat or cull lame sheep (*LC1 = typical "best practices"; LC2 = "slow to act"; LC3 = "slow to act, delayed culling"; for more details, see O'Kane et al. (2017) result section 3.3*)
Perrin *et al.* (2020)	Questionnaire made by Perrin *et al.* (2020)	2	Environment	Outdoor access	Evolution of full time grazing between the conversion beginning into organic farming and the time of the survey (which occurred at least 6.5 to 7 years after the conversion beginning)

1: Number of items related to the assessment of animal welfare in the questionnaire. **2:** Indicator of animal welfare used in the articles to describe potential relationships between the farmer well-being and the welfare of their animals.

Table 5: Synthesis of the methods used to assess animal welfare in the 16 reviewed studies with a quantitative approach included in a scoping review (aiming to map the methods used to describe —and compile pieces of evidence of— relationships between farmer well-being and animal welfare) (detailed version; part 3/3)

**Articles**	**Questionnaire, test or protocol**	**N_{Items}[1]**	**Dimension**	**Aspect of the dimension**	**Indicator used[2]**
Pol *et al.* (2021)	Questionnaire made by Pol *et al.* (2021)	7	Environment<br>Management	Housing enrichment, Housing type<br>Animal handling, Pain management, Procedures, Socialization	Three clusters of farms based on farmers' satisfaction with their work, and the implemented management practices (obtained after a multiple correspondence analysis followed by an ascendant hierarchical clustering)
Rilanto *et al.* (2022)	Questionnaire made by Rilanto *et al.* (2022)	2	Management	Culling	Culling rate; Mean age of culled cows; Culling rate as an active component in a k-mean clustering algorithm; Mean age of culled cows as an active component in a k-mean clustering algorithm
Spigarelli *et al.* (2021)	Protocol made by the European Food Security Authority (EFSA) Panel on Animal Health and Animal Welfare (2015)<br>Qualitative Behaviour Assessment described in the Welfare Quality protocol for dairy cattle (Welfare Quality project, 2009)	18<br>20	Health<br>Behaviour<br>Mental state	Body condition, Digestion, Lesions, Locomotion, Mortality, Reproduction, Respiration, Udder health<br>Reactivity<br>Attitude, Emotion	Overall animal welfare index as an active variable in a principal component analysis (0 - 100, where a higher score indicates a higher animal welfare state; *Sum of scores related to clinical observations and herd health data [scores were attributed according to quartiles: 1st quartile of a variable = score 1, 4th quartile = score 4. For more details regarding the index calculation, see Data Analysis section of Spigarelli et al. 2021*)
Vicic *et al.* (2022)	Questionnaire made by Vicic *et al.* (2022)	1	Management	Euthanasia	Euthanasia of non-replacement dairy calves (Yes, No)

1: Number of items related to the assessment of animal welfare in the questionnaire. **2:** Indicator of animal welfare used in the articles to describe potential relationships between the farmer well-being and the welfare of their animals.
